# Observation of high-temperature macromolecular confinement in lyophilised protein formulations using terahertz spectroscopy

**DOI:** 10.1016/j.ijpx.2019.100022

**Published:** 2019-07-08

**Authors:** Talia A. Shmool, P.J. Woodhams, Markus Leutzsch, Amberley D. Stephens, Mario U. Gaimann, Michael D. Mantle, Gabriele S. Kaminski Schierle, Christopher F. van der Walle, J. Axel Zeitler

**Affiliations:** a Department of Chemical Engineering and Biotechnology, University of Cambridge, Philippa Fawcett Drive, Cambridge CB3 0AS, United Kingdom; b Biopharmaceutical Development, AstraZeneca, Granta Park, Cambridge CB21 6GH, United Kingdom

**Keywords:** Lyophilisation, Protein stability, Structural dynamics, Glass transition, Thermal characterisation, Disorder, Vibrational spectroscopy, Terahertz spectroscopy

## Abstract

Characterising the structural dynamics of proteins and the effects of excipients are critical for optimising the design of formulations. In this work we investigated four lyophilised formulations containing bovine serum albumin (BSA) and three formulations containing a monoclonal antibody (mAb, here mAb1), and explored the role of the excipients polysorbate 80, sucrose, trehalose, and arginine on stabilising proteins. By performing temperature variable terahertz time-domain spectroscopy (THz-TDS) experiments it is possible to study the vibrational dynamics of these formulations. The THz-TDS measurements reveal two distinct glass transition processes in all tested formulations. The lower temperature transition, $T_{g,\beta }$, is associated with the onset of local motion due to the secondary relaxation whilst the higher temperature transition, $T_{g,\alpha }$, marks the onset of the α-relaxation. For some of the formulations, containing globular BSA as well as mAb1, the absorption at terahertz frequencies does not increase further at temperatures above $T_{g,\alpha }$. Such behaviour is in contrast to our previous observations for small organic molecules as well as linear polymers where absorption is always observed to steadily increase with temperature due to the stronger absorption of terahertz radiation by more mobile dipoles. The absence of such further increase in absorption with higher temperatures therefore suggests a localised confinement of the protein/excipient matrix at high temperatures that hinders any further increase in mobility. We found that subtle changes in excipient composition had an effect on the transition temperatures $T_{g,\alpha }$ and $T_{g,\beta }$ as well as the vibrational confinement in the solid state. Further work is required to establish the potential significance of the vibrational confinement in the solid state on formulation stability and chemical degradation as well as what role the excipients play in achieving such confinement.

## Highlights

• Structural dynamics in lyophilised protein formulations can be probed with terahertz spectroscopy and two glass transition processes, $T_{g,\alpha }$ and $T_{g,\beta }$, are observed.
• Vibrational confinement upon thermal activation is observed resulting in no detectable changes in secondary structure but strongly reduced the molecular mobility at temperatures above $T_{g,\alpha }$.
• The confinement was found to be strongly dependent on the formulation.
• We hypothesise that confinement is linked to conformational states with potential effects on physical and chemical stability of the biomolecule during storage.

## Graphical abstract

**Figure:**
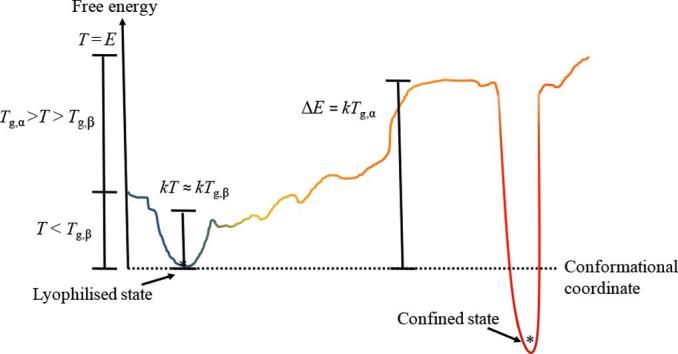

## 1 Introduction

Terahertz radiation can probe rotational translations, low-frequency bond vibrations, crystalline phonon vibrations, hydrogen-bond stretches, and torsional vibrations within a material (Sibik et al., 2014). Similar to low frequency Raman scattering, neutron scattering and light scattering techniques which cover a range of energy, frequency, and timescales, photon energies oscillating at terahertz frequencies (0.1–3 THz) can excite low energetic intermolecular interactions of materials, including phonon modes and hydrogen bond vibrations (Sibik and Zeitler, 2016). Recently, terahertz time-domain spectroscopy (THz-TDS) has been used to investigate the dynamics of the aqueous hydration shell of proteins at the terahertz region (Tielrooij et al., 2009). This is possible since the dielectric response corresponding to the reorientation of water dipoles has been shown to occur on picosecond timescales in the terahertz region (Tielrooij et al., 2009).

Understanding the mechanism of how a solid matrix can stabilise a dry-state protein is essential for developing predictive parameters against aggregation and degradation processes (Moorthy et al., 2015). In the literature, several studies into the dynamics of solid-state proteins examined the coupling of protein internal dynamics to the dynamic properties of a host matrix, and a selection of excipients which slowed down protein dynamics at low temperatures were identified (Cicerone et al., 2015, Markelz et al., 2007, Mizuno and Pikal, 2013). A number of dielectric relaxation processes have been described in the literature at least two of which are observed by all amorphous molecular materials, including proteins: the primary, or α-relaxation process, associated with large-scale mobility and the secondary, or β-relaxation, associated with local mobility, or small-scale mobility (Moorthy et al., 2015, Cicerone et al., 2015, Johari and Goldstein, 1970). Notably, the Johari-Goldstein (JG) β-relaxation process is considered a universal feature of all amorphous materials and is widely regarded to be dominated by the intermolecular degrees of freedom of a molecular material (Johari and Goldstein, 1970, Williams and Watts, 1971, Yu et al., 2013, Williams et al., 1955). The potential energy surface (PES) model proposed by Goldstein half a century ago can be used to understand the molecular dynamics in amorphous systems, and that intra- and intermolecular processes are always fundamentally coupled by means of the PES (Goldstein, 1969). Given that neutron scattering is sensitive to processes on the order of a nanoseconds or faster, one study utilised neutron scattering experiments in order to establish a link between the rate of protein degradation and the β-relaxation dynamics measured by the mean squared displacement, $\langle u^{2}\rangle$, for a series of lyophilised formulations (Cicerone and Soles, 2004). Specifically, the amplitude of the fast β-process, which has been associated with motions occurring on the timescale of picoseconds, is proportional to $\langle u^{2}\rangle$ (Cicerone and Soles, 2004, Cicerone et al., 2015). Numerous studies have been completed with the motivation of understanding the β-relaxation dynamics in order to utilise it as a parameter and a robust metric for predicting protein stability; however, the majority of these works have been completed using neutron back scattering, and these experiments cannot be implemented for routine formulation analysis due to time and cost limitations (Cicerone et al., 2015, Moorthy et al., 2015, Castellanos et al., 2017, Pearson and Smith, 1998). Dielectric spectroscopy which probes processes in the 10^−6^ and 10^−12^ s range and MHz/GHz frequency range, has been used to understand the dynamic relaxation processes of protein-solvent interfaces (Oleinikova et al., 2004). Techniques such as circular dichroism (CD) spectroscopy and X-ray crystallography are typically used to study the structure of proteins in solution and in crystalline solids. CD spectroscopy requires samples to be dissolved and crystallography is of limited use in studying the amorphous powders produced by lyophilisation (Mensink et al., 2017). Moreover, vibrational spectroscopy methods such as Fourier transform infrared spectroscopy (FTIR) and Raman spectroscopy can be used to probe the secondary structure of proteins after lyophilisation, and how they are affected by the presence or absence of surfactants and stabilising excipients. Nonetheless, dielectric spectroscopy and neutron scattering experiments are considered the optimal techniques for investigating protein stability given that these techniques are sensitive to larger time scales, and thus it is essential to investigate protein motions above and below the glass transition temperature ($T_{g}$) as these are thought to play a critical role in protein stability. Previously we have used THz-TDS and dynamic mechanical analysis (DMA) to measure the α- and β-relaxation dynamics of polymers (Shmool and Zeitler, 2019). While these transitions have been detected using DMA (Kissi et al., 2018), these are more noticeable in studies that utilise neutron back scattering (Buchenau et al., 1994), and terahertz time-domain spectroscopy (THz-TDS) (Markelz et al., 2007). Similar to neutron scattering, THz-TDS can be used to measure molecular mobility at the relevant length and timescales over a broad temperature range by probing molecular dipoles at frequencies of 0.1–3 THz. Using THz-TDS, at the glass transition temperature ($T_{g}$) we can observe the $T_{g,\alpha }$ associated with the α-relaxation process, and below $T_{g}$, we can observe $T_{g,\beta }$ associated with the β-relaxation process (Yu et al., 2013, Alegria and Colmenero, 2016, Cerveny et al., 2002, Roy and Inglefield, 1990, Barnes et al., 2001, Sibik and Zeitler, 2016). In doing so, THz-TDS can be used to characterise the molecular mobility of a system, which is a key factor when determining the chemical stability of a formulation, as an increase in molecular mobility has been directly linked to an increase in chemical degradation of a material and its storage stability (Shalaev et al., 2019). Thus, using THz-TDS it is possible to investigate the interplay between dielectric and vibrational dynamics and study the molecular mobility and structural dynamics of lyophilised formulations.

During formulation development, proteins are commonly lyophilised with sugars to produce dry therapeutic protein products in order to optimise their stability (Moorthy et al., 2015, Wang et al., 2009, Mensink et al., 2017). Using lyophilisation proteins can be dried to yield a lightweight product which can be easily preserved, shipped, and rehydrated (Moorthy et al., 2015, Mensink et al., 2017). In the lyophilisation process, a solution containing a protein is frozen, and subsequently ice is removed via sublimation, which can expose the protein to various stresses, such as denaturation at the ice surface, localised changes in pH, and the formation of new hydrogen bonds. Thus, lyophilisation requires a suitable organic molecular matrix to stabilise the protein of interest in the absence of protein molecules. The thermodynamic role of the matrix is to prevent degradation and provide stability to the protein while maintaining the native conformation of the protein as ice and bound water molecules are removed (Mensink et al., 2017, Roughton et al., 2013). Various studies have shown that non-reducing sugars, such as sucrose and trehalose, and non-ionic surfactants such as polysorbate 80 serve as effective stabilising agents (Mensink et al., 2017, Agarkhed et al., 2012). Additionally, excipients, for example L-arginine, have been used to increase solubility and minimise aggregation during lyophilisation (Moorthy et al., 2015).

The aim of this work is to understand the molecular mobility behaviour and structural dynamics of seven distinct protein excipient mixtures, and the dependance of each on temperature. We investigate the effects of different excipients, including sucrose, trehalose, L-arginine, and polysorbate 80 on each distinct formulation, with bovine serum albumin (BSA) as a model protein. We furthermore investigate the effects of adding polysorbate as well as altering the concentration on three monoclonal antibody (mAb) containing formulations. By comparing systems which contain the higher molecular weight and Y-shaped mAb versus globular BSA, we can investigate the effects of molecular weight and structure in the lyophilised formulations. We aim to provide a comprehensive understanding of the molecular dynamics of materials leading up to the glass transition temperature ($T_{g}$), and a relationship between the relaxation dynamics and the molecular structure of the lyophilised formulations.

## 2 Experimental methods

### 2.1 Materials

BSA, sucrose, glycine, histidine, L-histidine monohydrate, L-arginine, polysorbate 80 (poly-oxyethylene sorbitan mono-oleate) were all purchased from Sigma-Aldrich (Dorset, UK). The mAb used, mAb1, was obtained from AstraZeneca (Cambridge, UK) is a recombinant human IgG1 monoclonal antibody, with MW of approximately 144.8 kDa and extinction coefficient 1.42 mg (ml cm^−1^).

### 2.2 Lyophilised sample preparation

The seven different formulations are listed in Table 1. Additionally a sucrose/glycine mixture was prepared which contained 234 mM sucrose and 533 mM glycine. All the formulations were lyophilised using a lyophiliser (VirTis BenchTop, SP Industries Inc., Warminster, PA, USA), by the following steps: freezing was performed by cooling the shelf to 233 K at 160 mbar, and this temperature was maintained for 30 min. Primary drying was performed at 233 K at 133 mbar for 20 min, then the temperature was raised to 253 K at 133 mbar for 2440 min. This was followed by a secondary drying step at 313 K for 960 min, 133 mbar. The vials were subsequently closed under reduced pressure (of 266 mbar), at 298 K using a rubber stopper, and were crimped with aluminium seals. Vials were stored at 278 K until measurement and analysis. The water content for each lyophilised formulation was determined using Karl Fischer coulometric titration, ensuring that the residual moisture for each vial was less than 2.5%. The concentration of representative samples was measured in triplicate by UV-absorbance at 280 nm (A280) using a Trinean DropSense Multi-Channel Spectrophotometer (Unchained Labs, Pleasanton, California, USA).

**Table 1:** Composition of the formulations investigated in this study.

Formulation	Components
F1	2.3 mM BSA, 25 mM histidine-HCl, 265 mM sucrose
F2	1.1 mM BSA, 25 mM histidine-HCl, 265 mM sucrose
F3	1.5 mM BSA, 25 mM histidine-HCl, 130 mM trehalose, 50 mM arginine-HCl, 1.7 μM polysorbate 80
F4	1.1 mM BSA, 25 mM histidine-HCl, 265 mM sucrose, 3.3 μM polysorbate 80
F5	0.28 mM mAb1, 25 mM histidine-HCl, 205 mM sucrose
F6	0.49 mM mAb1, 25 mM histidine-HCl, 265 mM sucrose
F7	0.49 mM mAb1, 25 mM histidine-HCl, 265 mM sucrose, 3.3 μM polysorbate 80

### 2.3 THz-TDS sample preparation

The samples were prepared in a glove bag (AtmosBag, Sigma-Aldrich, Dorset, UK) which was purged with dry nitrogen gas (relative humidity < 1%) to avoid moisture sorption from atmospheric water vapour. The lyophilised powder samples were pressed into 13 mm diameter flat-faced pellets, using a load of 1.5 metric tons. The resulting pellets were between 300 and 700 μm in thickness and 70 mg in weight each, and were placed between two-quartz windows. This sandwich structure was sealed in the sample holder, as described previously (Shmool and Zeitler, 2019).

### 2.4 Circular dichroism spectroscopy (CD)

Circular dichroism spectroscopy (CD) was used to investigate the structural changes of the following formulations: F2 at 278 K and after being heated to 370 K (following a THz-TDS experiment); F4 at 278 K and after being heated to 410 K in glass vials in a dry heat block. We also analysed BSA powder (A7906, Sigma-Aldrich) as received from the supplier. All BSA formulations were dissolved in dH_2_O and diluted to a concentration of 3–4 μm before analysis by CD. Protein concentration was calculated by measuring absorbance at 280 nm on a Nanovue spectrophotometer (GE Healthcare, Uppsala, Sweden) using the extinction coefficient of 43,824 M^−1^ cm^−1^ (Gill and von Hippel, 1989).

For formulations containing mAb1, F6 was analysed at 278 K and after being heated to 330 K. F7 was analysed at 278 K and after being heated to 370 K in glass vials in a dry heat block. All mAb1 formulations were dissolved in dH_2_O and diluted to a concentration of 1 μm, calculated using the extinction coefficient of 207,360 M^−1^ cm^−1^ (Pan et al., 2018).

Samples were analysed in a 1 mm cuvette at a temperature of 303 K. CD spectra were acquired using a JASCO J-810 spectropolarimeter (Jasco Inc., Easton, MD, USA). Spectra were recorded over the spectral range of 250–190 nm, with a resolution of 0.5 nm, a continuous scan at 50 nm min^−1^, and a bandwidth resolution of 1 nm. 10 accumulations were obtained for each sample and each experiment was repeated three times. CD spectra of dH_2_O were recorded and subtracted from each sample spectrum. Mean residue ellipticity was calculated using Eq. (1), where $\left[\theta \right]$ is the mean residue ellipticity (°cm^2^ dmol^−1^), $\theta_{\mathrm{obs}}$ the observed ellipticity, *l* the path length (mm), *c* the molar concentration (M) and *n* the number of residues (583 amino acids for BSA and 1330 for mAb1).

(1) $$[\theta ]=\frac{\theta_{\mathrm{obs}}}{\mathrm{lcn}}$$

### 2.5 Fourier transform infrared spectroscopy (FTIR)

FTIR was used to examine the change in the secondary structure of neat lyophilised BSA powder at 278 K. F2 was analysed at 278 K and after being heated to 370 K. F4 was analysed at 278 K and after being heated to 410 K. F6 was analysed at 278 K and after being heated to 330 K. F7 was analysed at 278 K and after being heated to 370 K.

For FTIR analysis of the protein, 300 μg of the protein were mixed with potassium bromide (KBr) using an agate mortar, and pressed into 7 mm diameter self-supporting disks using a load of 10 tons. FTIR spectra were acquired using a Cary 680 FTIR spectrometer (Agilent Technologies Inc., Santa Clara, CA, USA) by co-averaging 120 scans and at a resolution of 1 cm^−1^. At least four spectra were measured for each formulation. The FTIR spectra of the pure excipients were recorded and subtracted from each sample spectrum, by a linear subtraction scaled to the excipient peak at 851 cm^−1^. The recorded spectra were normalised based on the total area under the curve. To estimate the secondary structure composition, each spectrum was smoothed using a Savitsky-Golay derivative function fitted over 14 cm^−1^ and the second derivative of the amide I region from 1590 to 1620 cm^−1^ was calculated. Ten Gaussian peaks were fitted to the second derivative spectrum and the ratio of the peak areas compared to the total area under the curve was used to estimate the secondary structure content (Yang et al., 2015).

### 2.6 Solid-state nuclear magnetic resonance spectroscopy (ssNMR)

Solid-state nuclear magnetic resonance spectroscopy was used to investigate the structural changes of the unheated (278 K) and heated (370 K) samples of F2, unheated (278 K) and heated (410 K) samples of F4, unheated (278 K) and heated (330 K) samples of F6, and unheated (278 K) and heated (370 K) samples of F7. ^13^C CP-MAS spectra were acquired using a Bruker AVANCE 400 (Bruker UK Limited, Coventry, UK) equipped with a Bruker 4 mm CP/MAS ^1^H/X BB probe at room temperature, operating at a magic angle spinning (MAS) rate of 12 kHz together with the SPINAL64-proton decoupling pulse sequence (Fung et al., 2000) during acquisition. The cross polarisation (CP) efficiency was optimised using a glycine sample. For cross polarisation a ramped CP from 50 to 100% with a contact time of 2 ms was used. Data were acquired by averaging 1200 free induction decays containing 2048 complex data points with a total acquisition time of 25 ms and a relaxation delay of 5 s between individual scans unless indicated otherwise. Proton spin-lattice relaxation times $T_{1}$ were determined by inversion recovery with ^13^C detection via CP. For each sample eight individual time points *t* were acquired (0.01, 0.05, 0.1, 0.2, 0.5,1.0, 2.0 and 5.0 s).

Proton spin-relaxation times in the rotating frame $T_{1\rho }$ were determined by varying the ^1^H spin lock pulse time $t_{\mathrm{SL}}$ following a π/2 ^1^H pulse with ^13^C detection via CP. For each sample eight individual time points *t* were acquired (0.1, 1, 5, 10, 20, 30, 40 and 50 ms).

To obtain $T_{1}$ and $T_{1\rho }$ values of the BSA and the sucrose, the spectra were integrated with the T1T2 module in Topspin 3.5pl7 (Bruker Biospin Corporation, Billerica, MA, USA) in the carbonyl region (165–185 ppm) and the alcohol and anomeric signal region (67–102 ppm) to obtain information from the components respectively. The data were then exported to MATLAB (R2016b, The MathWorks Inc., Natick, MA, USA) and fitted to the following equations:

(2) $$M(t)=M_{0}(1-2{\mathrm{Ae}}^{\frac{-t}{T_{1}}})$$

(3) $$M(t_{\mathrm{SL}})=M_{0}e^{\frac{-t_{\mathrm{SL}}}{T_{1\rho }}}$$

where M(t) is the magnetisation at a given time point $t,\;M_{0}$ the magnetisation at equilibrium and *A* is a correction factor. ^13^C spectra were externally referenced to the methylene signal of adamantane (*δ* = 38.48 ppm).

### 2.7 THz-TDS experimental setup and data analysis

The THz-TDS spectra were acquired using the methodology introduced previously (Shmool and Zeitler, 2019). In order to calculate the absorption coefficient and the refractive index of the sample a modified method for extracting the optical constants from terahertz measurements based on the concept introduced by Duvillaret et al. was used (Duvillaret et al., 1996, Sibik and Zeitler, 2015). The changes in dynamics of the samples were analysed by investigating the change in the absorption coefficient at a frequency of 1 THz as a function of temperature using the methodology introduced by Shmool and Zeitler (2019).

### 2.8 Modulated differential scanning calorimetry (MDSC)

A Q2000 Differential Scanning Calorimeter (TA Instruments, New Castle, DE, USA) was used to determine the calorimetric glass transition temperature ($T_{g,\mathrm{DSC}}$, defined by the onset temperature) for each material. 2–3 mg of sample material were placed in hermetically sealed aluminium pans under a constant flow nitrogen atmosphere (flow rate 50 ml min^−1^) and cooled at a rate of 3 K min^−1^ from room temperature to approximately 243 K. The samples were subsequently heated at a rate of 10 K min^−1^ to 295 K and then at 5 K min^−1^ to 373 K. The modulation frequency was 0.006 K s^−1^. The temperature and heat flow of the instrument were calibrated using indium ($T_{m}=430$ K, $\Delta H_{\mathrm{fus}}=29\;J\;g^{-1}$).

## 3 Results and discussion

### 3.1 Terahertz time-domain spectroscopy (THz-TDS)

The terahertz spectra of all the formulations showed an increase in absorption with frequency and temperature (see ESI), and no discrete spectral features were present over the entire investigated range, in line with previous measurements of amorphous molecular solids (Sibik et al., 2014). We chose the frequency of 1 THz to further investigate the relationship between the increase of absorption coefficient and temperature, as the signal-to-noise ratio of the measurement at 1 THz is high and we previously have shown that the frequency is suitable to follow the dynamics of amorphous systems (Sibik and Zeitler, 2015).

**Fig. 1:**
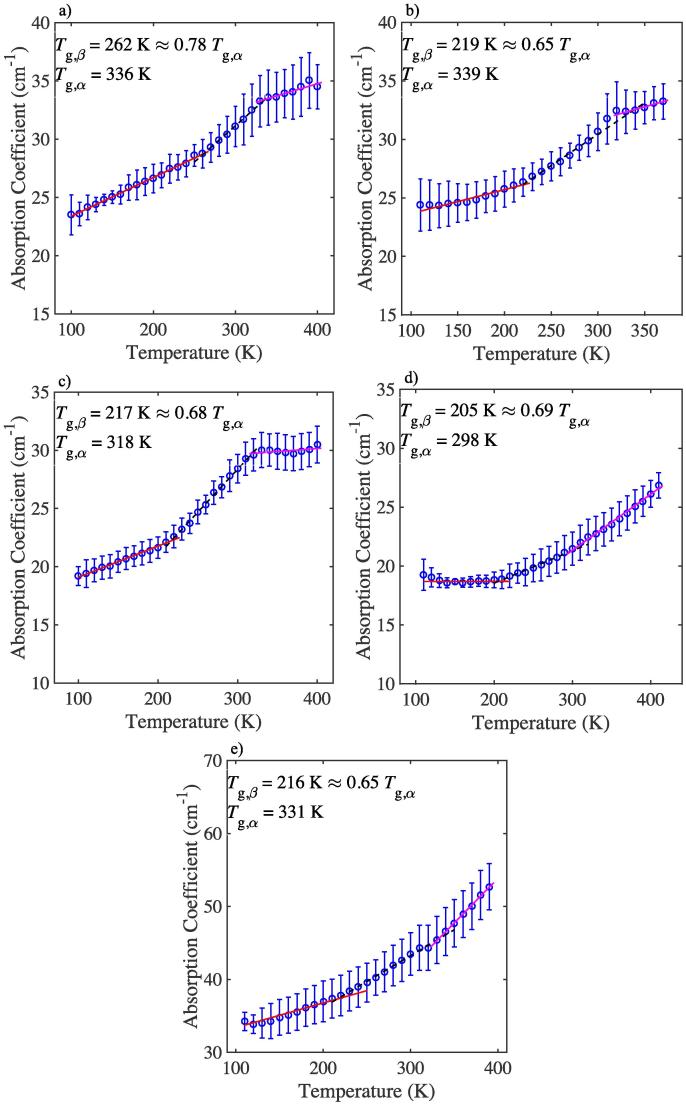
Mean terahertz absorption coefficient as a function of temperature at 1 THz for BSA formulations. a) F1, b) F2, c) F3, d) F4 and e) BSA. Lines show the linear fits for the three different regions. Error bars represent the standard deviation for *n* samples: n=3.

**Fig. 2:**
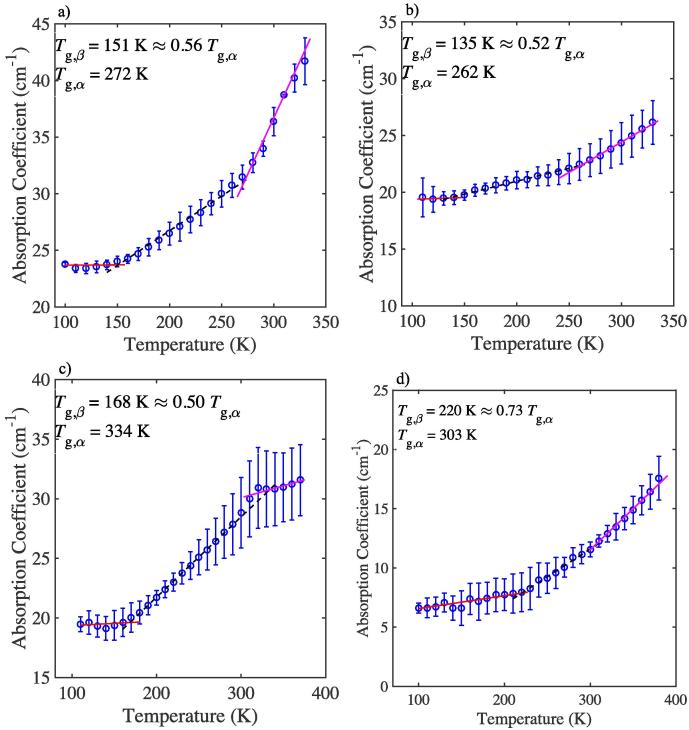
Mean terahertz absorption coefficient as a function of temperature at 1 THz for mAb1 formulations. a) F5, b) F6, c) F7, and d) a sample of a lyophilised mixture of sucrose and glycine. Lines show the different linear fits of the respective regions. Error bars represent the standard deviation for *n* samples: n=3 for a), c), and d), and n=4 for b).

The changes in absorption at a frequency of 1 THz with temperature for the formulations are plotted in Fig. 1, Fig. 2. In line with previous experiments for a range of organic molecular materials, three distinct temperature regions can be identified for each material and $T_{g,\beta }$ was defined as the intersection point of the two best-fit linear fits at low temperatures, and $T_{g,\alpha }$ was defined as the intersection point of the two best-fit linear lines at high temperatures (Sibik et al., 2014, Mensink et al., 2017, Shmool and Zeitler, 2019). It is worth noting that the values of $T_{g,\alpha }$, as determined from the THz-TDS experiments, are in good agreement with our own calorimetric measurements, $T_{g,\mathrm{DSC}}$, as well as the values reported in the literature for these materials (Table 2, see ESI) (Duddu and Dal Monte, 1997, Srirangsan et al., 2010, Wang et al., 2009).

**Table 2:** Gradient, *m*, of the linear fit (y=mx+c) for the respective temperature regions as outlined by Shmool and Zeitler (2019) as well as the respective transition temperatures determined based on the terahertz analysis. For all samples three regions were identified using the data analysis routine.

Formulation	*m*, Region 1	*m*, Region 2	*m*, Region 3	$T_{g,\beta }$	$T_{g,\alpha }$	$T_{g,\mathrm{DSC}}$
	(cm^−1^ K^−1^)	(cm^−1^ K^−1^)	(cm^−1^ K^−1^)	(K)	(K)	(K)
F1	0.033±0.00059	0.062±0.0018	0.021±0.0036	262	336	310
F2	0.021±0.0020	0.054±0.0028	0.023±0.0027	219	339	311
F3	0.027±0.0091	0.074±0.0016	0.0051±0.0028	217	318	302
F4	0.00014±0.0017	0.028±0.0011	0.048±0.0014	205	298	305
Neat BSA	0.034±0.0031	0.071±0.0017	0.12±0.0023	216	331	N/A
Sucrose and glycine mixture	0.011±0.0014	0.047±0.0021	0.070±0.0018	220	303	N/A
F5	0.00056±0.0050	0.061±0.0020	0.020±0.011	151	272	282
F6	0.0033±0.0034	0.023±0.00087	0.053±0.0021	135	262	297
F7	0.0038±0.0047	0.067±0.0011	0.020±0.0046	168	334	304

#### 3.1.1 Understanding the change in molecular dynamics using THz-TDS

In the literature, the transition temperature at which internal protein mobility occurs upon heating from low temperatures has been referred to as the protein dynamical transition temperature (Mizuno and Pikal, 2013, Markelz et al., 2007). However, there is some controversy regarding the specific motions and relaxation processes that are associated with this dynamical transition. For example, using THz-TDS, protein systems of oxidised cytochrome c solutions and lyophilised horse heart cytochrome c, showed a sharp increase in the linear temperature dependence of the terahertz dielectric response at 200 K (Markelz et al., 2007). This was attributed to activated side-chain motions which are involved in the dynamical transition. It is important to highlight in this context that there is some evidence to suggest that the temperature of the dynamics transition is frequency dependent and also conditional on the presence of water, although both aspects are still relatively poorly understood and contrasting observations appear to have been reported (Khodadadi et al., 2008, Khodadadi and Sokolov, 2015, Khodadadi and Sokolov, 2017, Bellissent-Funel et al., 2016).

We have previously shown in our own work that local dipole mobility is associated with the onset of $T_{g,\beta }$, regardless of whether these motions originate from side groups or larger domains purely depending on the height of the respective energy barriers (Sibik and Zeitler, 2016, Shmool and Zeitler, 2019). We also showed that this appears to be universal for all organic molecular materials that we have investigated to date including polyalcohols (Sibik et al., 2013, Sibik et al., 2014, Sibik and Zeitler, 2015, Ruggiero et al., 2017), pharmaceutical drugs (Sibik et al., 2015, Sibik and Zeitler, 2016, Kissi et al., 2018), proteins (Sibik and Zeitler, 2016, Mensink et al., 2017) and polymers (Shmool and Zeitler, 2019). Ngai et al. recently highlighted the same phenomenon for a further set of protein samples by carefully re-analysing data previously published in the literature using a range of techniques (Ngai et al., 2019). Furthermore, the experiments of Mizuno and Pikal reported the observation of an endothermic pre-$T_{g}$ event in the DSC thermogram when heating lyophilised proteins around 310–330 K and attributed this event also to the onset of protein internal dynamics. However the authors linked these motions to the α-relaxation process rather than the β-relaxation (Mizuno and Pikal, 2013, Sibik and Zeitler, 2016, Shmool and Zeitler, 2019). Frontzek et al. also report a glass-like transition process in dry protein at temperatures similar to $T_{g,\beta }$ (Frontzek et al., 2014). The significance of understanding the onset of the mobility of protein molecules has been highlighted repeatedly as the key to understanding protein stability. Specifically, Mizuno and Pikal suggested a link of the activation of protein motions associated with the α-relaxation with initiating protein degradation (Mizuno and Pikal, 2013). Furthermore, the so-called ‘$T_{g}-50$ K rule’ has been proposed more widely in the pharmaceutical field as a general rule that a given material should be stored at least at 50 K below its $T_{g}$ (Mizuno and Pikal, 2013).

We have previously shown that the different molecular motions of a material can be tracked with temperature using THz-TDS (Sibik and Zeitler, 2016, Shmool and Zeitler, 2019). At low temperatures, in the glassy state, it is widely accepted that the motions of the system are restricted and confined to its local potential energy minimum. We define $T_{g,\beta }$ as the temperature threshold at which the molecules have sufficient free volume and energy to escape their lowest energy configurational barrier and explore different conformational environments as the temperature increases further. Upon exceeding $T_{g,\alpha }$, the glass transition temperature, the mobility of the molecules can either: (1) continue to increase gradually, as the molecules keep on with exploring different conformational environments due to their inherent flexibility; or, (2) the molecular mobility of the system plateaus when the molecules happen to sample a sufficiently low energy conformation that is so stable that the molecule becomes trapped in an energy minimum that exceeds the available thermal energy. The slope *m* (Table 2), corresponding to the changes in absorption coefficient with temperature, is directly linked to the molecular mobility of the system (Shmool and Zeitler, 2019). For all the formulations that we have investigated we observed that the linear gradient in the region of $T<T_{g,\beta }$ is typically lower compared to $T_{g,\beta }<T<T_{g,\alpha }$. At temperatures above $T_{g,\alpha }$ we can broadly distinguish two types of observation for the change in absorption with temperature: either the gradient increases further (expected behaviour at high temperatures) or it decreases relative to the region of $T_{g,\beta }<T<T_{g,\alpha }$ and remains relatively flat at high temperatures (indicating confinement of the molecular mobility).

F1, F2, and F3 all show a shallow gradient at temperatures below $T_{g,\beta }$ as well as above $T_{g,\alpha }$ (shown in Fig. 1). Previously we have always observed an increase in terahertz absorption with temperature above $T_{g,\alpha }$ for a wide range of less complex organic molecular materials due to their steadily increasing molecular mobility (Sibik and Zeitler, 2016). We hypothesise that in these systems the local conformations of the BSA molecules become trapped in the matrix by energy minima that are relatively more stable than *kT* (Khodadadi and Sokolov, 2017). As a result, the motions of the most flexible domains of the BSA molecules and their matrix become restricted. Such confinement reduces the flexibility and hence molecular mobility of the protein and matrix molecules. A plateau in terahertz absorption at high temperatures could therefore be interpreted as a molecular confinement of the protein in its surrounding matrix.

When comparing the behaviour of the different formulations, we found that $T_{g,\beta }(F1)>T_{g,\beta }(F2):262$ vs. 219 K. Both F1 and F2 were measured to have similar values of $T_{g,\alpha }$ and both exhibit restricted mobility above 340 K. Whilst the difference in relative amounts of histidine and sucrose compared to BSA clearly has a strong effect on the onset of mobility, it does not affect the large-scale motions at $T_{g,\alpha }$ (336 vs 339 K). In both cases the system exhibited confinement of the protein in matrix at high temperatures, yet, in the absence of histidine and sucrose there was no evidence of any such confinement at temperatures above $T_{g,\alpha }$ (Fig. 1d). The difference between the formulations is that in F1 there is less sucrose and histidine available relative to BSA compared to F2. As a result of the lower amount of stabilising excipients, BSA is more likely to aggregate in F1 prior to, and during, lyophilisation. Increased aggregation results in a significant reduction of local mobility, reflected in a higher $T_{g,\beta }$, due to the stronger local protein interactions in the absence of stabilising excipients (Moorthy et al., 2015, Cicerone et al., 2015, Mensink et al., 2017). In the case of more abundantly available sucrose and histidine the individual protein molecules can stabilise their conformations by forming weaker and more flexible hydrogen bonds with the excipients. Given that these protein-excipient interactions must be weaker compared to the protein-protein interactions we consider it likely that the difference in terahertz dynamics which are observed in our experiments are dominated by the protein conformations themselves rather than by the excipients or protein interactions.

**Fig. 3:**
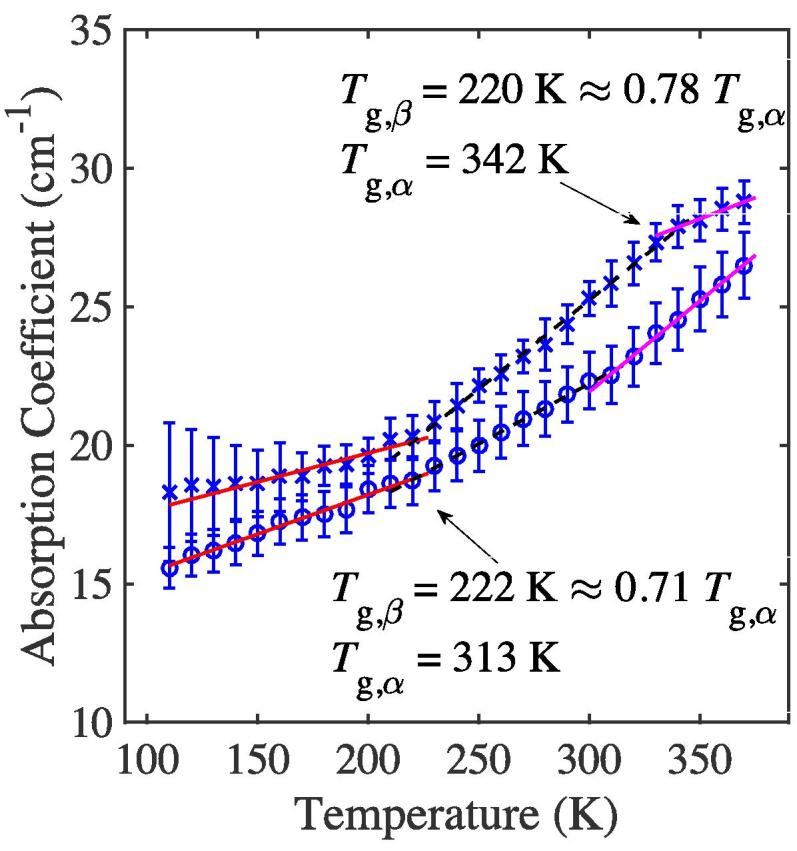
Terahertz absorption as a function of temperature at 1 THz for F2 in two subsequent heating cycles. The ‘×’ markers represent the data for Cycle 1 of samples heated at 10 K increments, and the ‘∘’ markers represent the data for Cycle 2 of the same sample, which was quench cooled in situ and under vacuum within the cryostat following Cycle 1 and reheated at 10 K increments. Lines show the linear fits for the three thermal regions. Error bars represent the standard deviation for n=2 samples.

We further investigated the confinement of BSA in the matrix at high temperatures by subjecting a sample of F2 to a cycle of heating, subsequent rapid cooling followed by a final heating step in order to establish whether we can observe a hysteresis using THz-TDS. Our results show that there is a change in the molecular mobility behaviour of the material during the second heating cycle. The data in Table 2 and Fig. 3, show higher values of *m* at $T\geq T_{g,\alpha }$ in the second heating experiment compared to m≈0 for $T\geq T_{g,\alpha }$ in the first heating cycle. The results would suggest that at $T\geq T_{g,\alpha }$ during the first heating cycle, the BSA molecules end up in a confined state that corresponds to a local energy minimum on the PES. However, upon cooling, the conformations appear to change sufficiently that repeated heating from 100 K results in a different trajectory on the PES that does not end up in the confined state at $T\geq T_{g,\alpha }$.

Qualitatively, F3, which in addition to histidine also contains trehalose, instead of sucrose, and arginine and polysorbate, behaves similarly to F1 and F2 at terahertz frequencies (Fig. 1). In solution, excipients, such as arginine, have been shown to inhibit aggregation via interaction with the protein surface (Ratanji et al., 2014, Kim et al., 2014). Trehalose is also commonly used to stabilise proteins during lyophilisation and is thought to hinder aggregation via hydrogen bonding between sugar and protein (Moorthy et al., 2015). As evident from the THz-TDS data for F3, the changes in gradient are more defined and the values of $T_{g,\beta }=217$ K and $T_{g,\alpha }=318$ K are lower compared to F1 and F2. This could be due to the stabilising effects of the excipients, as discussed above. Notably, it would be useful to perform dynamic light scattering and size exclusion chromatography experiments on each sample in order to measure the size distribution and the percentage of aggregates present for the respective formulations (Stephens et al., 2018). This would determine whether there is a clear correlation between the proportion of aggregation of a given formulation and the observance of a plateau in the THz-TDS data.

As previously mentioned, sugars such as sucrose have been shown to stabilise proteins and mAbs in the solid state (Mensink et al., 2017). When considering the mechanism by which sugars stabilise a mAb we focus on the global and local mobility of the mAb and it is important to consider the molecular flexibility of the sugar as well as the effects of free volume. With increasing number of water molecules removed during the lyophilisation process the hydroxyl groups of the sugar form hydrogen bonds with the mAb and substitute the hydrogen bonds between the protein and water effectively forming a support structure for the mAb (Ohtake et al., 2011, Wang et al., 2009). As the hydrogen bonds are replaced, it is possible, and indeed likely, that the native structure of the mAb is not faithfully maintained and its hydrogen bonding network can change (Ohtake et al., 2011, Mensink et al., 2017, Murayama and Tomida, 2004). We again observe a higher value of $T_{g,\beta }$ for F5 compared to F6, similar to what we observed for BSA in F1 and F2 (which differed in the increased concentration of stabilising sucrose, Table 1). Specifically, in F5, there is a larger amount of sucrose relative to mAb1 molecules compared to in F6. Thus, the relative amount of hydrogen bonding between sucrose and mAb1 is greater in F5 compared to formulation F6. Therefore, the mAb1 molecules in F5 would be rigidly stabilised by the sucrose molecules, which reduce the free volume of the system and raise the value of $T_{g,\beta }$ and the barrier to mobility. We observe a higher $T_{g,\beta }=151$ K and $T_{g,\alpha }=272$ K for F5 compared to $T_{g,\beta }=135$ K and $T_{g,\alpha }=262$ K for F6 (Table 2). Moreover, a large separation and steep gradient between $T_{g,\beta }$ and $T_{g,\alpha }$ could suggest that the mAb1 molecules assemble a larger number of conformations, and specifically in F5, the mAb1 molecules adopt a greater number of conformational states, compared to the mAb1 molecules of F6 which exhibit a shallower gradient and a smaller temperature difference between $T_{g,\beta }$ and $T_{g,\alpha }$.

Notably, to investigate the effects of the addition of mAb1 on the values of $T_{g,\beta }$ and $T_{g,\alpha }$, we first studied a stable model system, which is routinely used in protein formulations: an excipient mixture of sucrose/glycine (Kasraian et al., 1998). The sucrose/glycine hydrogen bonded network exhibits values of $T_{g,\beta }=220$ K and $T_{g,\alpha }=303$ K (Fig. 2d), that are significantly higher than those observed for F5, F6, and F7 (Table 2). This suggests that when mAb1 is added to a formulation it creates a more loosely bound hydrogen bonded network, with more free volume available and reduced $T_{g,\beta }$ and $T_{g,\alpha }$ values, compared to the more strongly bound hydrogen bonded network of sucrose and glycine. This further supports that the local and large-scale mobility of a system are sensitive to the strength of the hydrogen bonding network and the free volume effects.

Polysorbate 80 has been widely used in the biopharmaceutical industry to reduce agitation-induced aggregation by protecting the protein from interface induced stresses which can be caused upon lyophilisation (Arakawa and Kita, 2000, Ratanji et al., 2014). The mechanism by which polysorbate 80 acts is still under debate, however, given its widespread use as a stabilising excipient its effects on the dynamics of formulations are of interest to us. We examine the effect of the surfactant polysorbate 80 on BSA in F4 and on the mAb in F7. The THz-TDS data of F4 exhibit a continuous increase in absorption coefficient with temperature, which is indicative of a continuous increase in molecular mobility (Fig. 1). It is worth highlighting that the values of $T_{g,\beta }$ and $T_{g,\alpha }$ are lower in F4 than for all other BSA formulations. It is possible that the presence of the polysorbate 80 molecules results in reduced local barrier heights and hence allow for higher low temperature mobility in F4 compared to the other formulations. Rather than only affecting the local barriers, and with that the value of $T_{g,\beta }$, the presence of polysorbate 80 also results in increased large-scale mobility, and hence lower values of $T_{g,\alpha }$. This effect is evident in both BSA formulations that contain polysorbate 80 (F3 and F4) and appears to be concentration dependent: the higher the concentration of polysorbate 80 the lower $T_{g,\alpha }$ (Table 2). Additionally, in contrast to F5 and F6, where the absorption coefficient continuously increases with temperature (Fig. 2), F7 exhibits confinement of motion at temperatures above $T_{g,\alpha }$, similar to the BSA formulations, F1-3. It is interesting to note that in the presence of polysorbate 80, F7 exhibits higher values of $T_{g,\beta }$ and $T_{g,\alpha }$ compared to F5 and F6. This is opposite to what we observed for the BSA formulations. Such a result is in agreement with the hypothesis that in the presence of polysorbate the hydrogen bonded network of the mAb is altered, as mAb1 molecules may bind more strongly to each other than to the interface during lyophilisation (Couston et al., 2013, Agarkhed et al., 2012). The increase in binding strength between the mAb1 molecules would raise the activation energy barrier for the onset of motions, as indicated by higher values of $T_{g,\beta }$ and $T_{g,\alpha }$. Moreover, the difference in behaviour of F4 compared to F7 could be due to the different surface characteristics of globular BSA versus the Y-shaped mAb. This could suggest that in the case of BSA, the surfactant molecules interact with the BSA molecules and destabilise the hydrogen bonded network in the solid matrix, however in the case of the mAb the surfactant molecules stabilise it via linking of adjacent hydrophilic and hydrophobic domains within the mAb.

From the above it is evident that upon heating, BSA or mAb in their respective excipient matrix can undergo conformational changes in structure. The composition of the excipient matrix can dictate the strength of these conformational changes, and the relative number of changes is represented by the steepness of the region 2 gradient of the THz spectra. This is dependant on the excipients used and the concentrations added, which influence whether a stronger scaffold is formed for the protein in the dry state. It can be suggested that a strong scaffold can be linked to systems which exhibit a plateau, and for which the protein is confined within the matrix at high temperatures. In contrast, some protein formulations exhibit an increase in absorption. For these the dynamics of the systems exhibit similar behaviour to that of small organic molecules, for which at higher temperatures the vibrational motions of the molecules increases continuously with no confinement of the protein in matrix taking place.

#### 3.1.2 Fourier transform infrared spectroscopy (FTIR) and circular dichroism (CD) spectroscopy

**Fig. 4:**
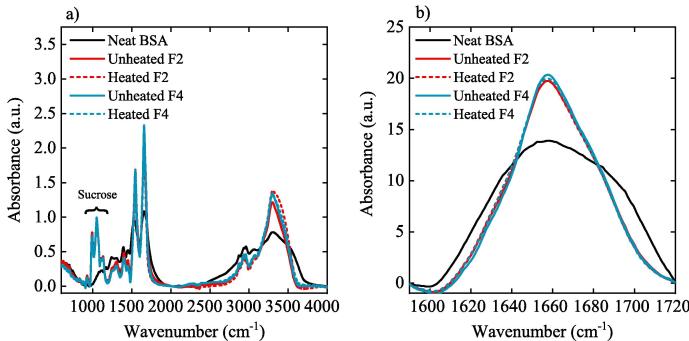
a) FTIR spectra, b) the amide I region of BSA. Black curve represents neat BSA, solid red curve represents F2, dashed red curve represents heated F2, solid blue curve represents F4, and dashed blue curve represents the heated F4. Spectra of the excipients have been subtracted from each spectra. (For interpretation of the references to colour in this figure legend, the reader is referred to the web version of this article.)

FTIR and CD spectroscopy were used to screen for gross structural changes in the BSA formulations before and after heating and following the addition of polysorbate 80. No significant changes were observed in the peak positions or intensities in the FTIR spectra (Fig. 4) or the CD spectra (Fig. S1), when either the formulations with or without polysorbate 80 were heated. The FTIR spectrum of neat BSA was in good agreement with that presented in the work of Qing et al. (1996). Significant differences were observed between the lyophilised BSA formulations and the samples of neat BSA as received from the supplier. The lyophilised formulations included additional peaks in the region 900–1200 cm^−1^, which were attributed to sucrose (Huang et al., 2017). Notably, the interaction between the excipients and the protein under investigation, can cause a shift in the protein peak positions and shapes, and thus a linear excipient subtraction will reduce the effects of the excipients on the spectrum, yet will not completely remove all the excipient related peaks.

FTIR measurements of the neat BSA showed an α-helix content of 15 ± 8% while the samples of F2 (both unheated and heated) contained 36 ± 10% and 41 ± 13% α-helix respectively. Additionally, FTIR measurements of the unheated BSA sample of F4 showed that it contains a higher content of α-helix compared to the neat BSA sample (40 ± 11% vs. 15 ± 8%) (see ESI), supporting that lyophilisation and the addition of excipients resulted in a conformational change to BSA which THz-TDS can detect.

The CD spectra of all BSA formulations showed negative bands at 222 nm and 208 nm and a positive band at 193 nm, characteristic of a high α-helical content (see ESI) (Takeda et al., 1989). The amide I band (1700–1600 cm^−1^) of the FTIR spectrum showed an asymmetric feature centred at approximately ∼1658 cm^−1^, (Fig. 4b), which is characteristic of a high α-helical content (Barth, 2007).

**Fig. 5:**
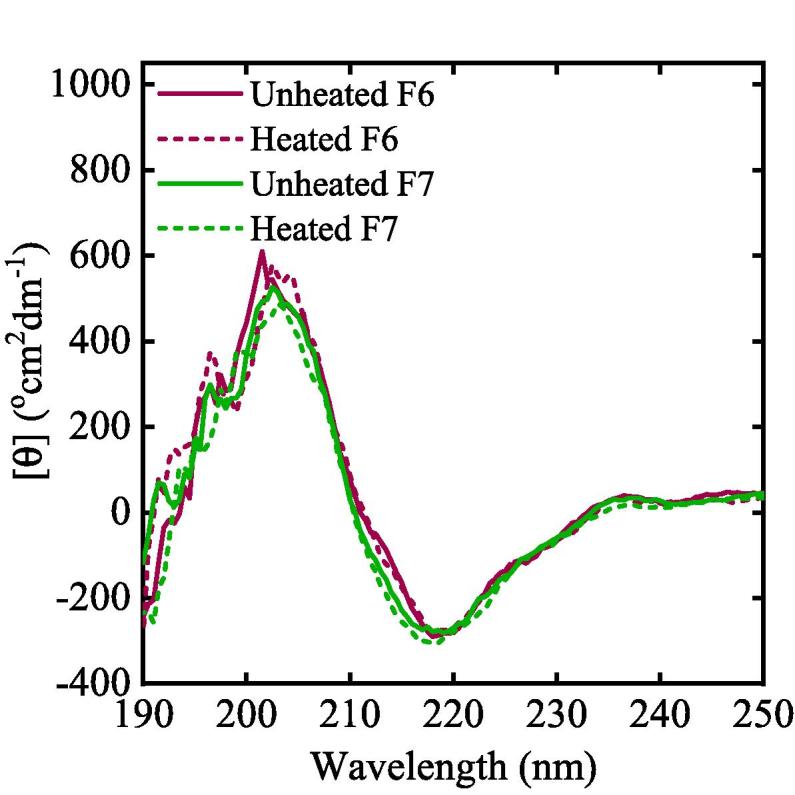
CD spectra of the two different mAb formulations. Solid and dashed pink curves represent unheated and heated F6 respectively, solid and dashed green curves represent heated and unheated F7 respectively. (For interpretation of the references to colour in this figure legend, the reader is referred to the web version of this article.)

**Fig. 6:**
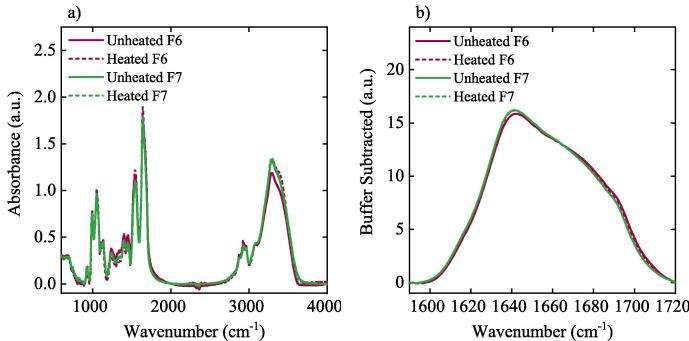
FTIR spectra of the a) whole spectral region and b) the amide I region of the two different mAb1 formulations. Solid and dashed pink curves represent unheated and heated F6 respectively, solid and dashed green curves represent heated and unheated F7 respectively. Buffers have been subtracted from each spectra. (For interpretation of the references to colour in this figure legend, the reader is referred to the web version of this article.)

No significant changes were observed upon heat treatment of the mAb formulations in the peak positions or intensities in the CD (Fig. 5) or FTIR spectra (Fig. 6) (see ESI).

The CD spectra for the F6 and F7 mAb formulations exhibited a typical spectra with a broad negative band at 218 nm characteristic of beta-sheet secondary structure (Joshi et al., 2014). Similarly, the amide I band of the FTIR spectrum showed an asymmetric band centred at ≈1640 cm^−1^, with shoulders at approximately 1670 cm^−1^ and 1690 cm^−1^ (Fig. 6b), which is characteristic of the high β-sheet content, estimated to be for example 56 ± 8% for unheated F6 (see ESI) (Barth, 2007).

#### 3.1.3 Solid-state nuclear magnetic resonance spectroscopy (ssNMR)

Solid-state NMR is a well established method for studying protein formulations (Yoshioka et al., 2011, Mensink et al., 2017, Mensink et al., 2016).

**Fig. 7:**
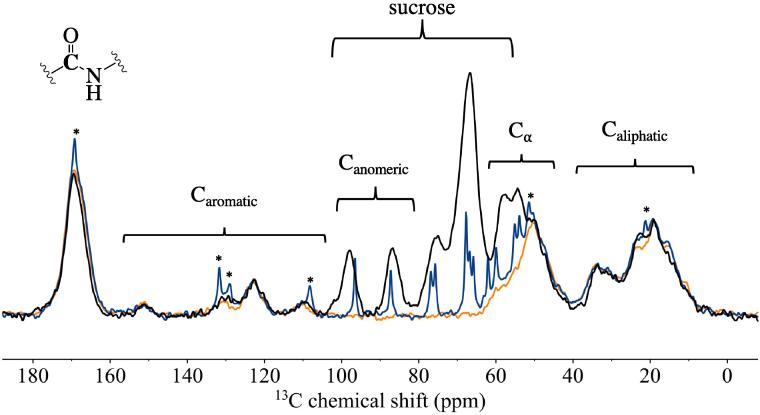
^13^C CP-MAS NMR spectra of F2 (), a physical mixture of the formulation components () and pure BSA (). All the spectra were normalised to the same carbonyl signal intensity. Data was obtained after averaging 1200 individual FIDs, for pure BSA and the physical mixture only 600 FIDs were averaged.

To investigate the structural changes on the molecular scale, ^13^C CP-MAS spectra of the BSA formulations before and after heating, and with the addition of 0.04% polysorbate 80 were acquired and compared to pure BSA and a physical mixture of the formulation components (for the THz-TDS spectra of the physical mix see ESI). Fig. 7 shows the ^13^C data obtained from neat BSA, F2 and a physical mixture of the F2 ingredients. The ^13^C NMR signals from amides (165–185 ppm), the ${\text{C}}_{\alpha }$ atoms (45–60 ppm), and aromatic (110–150 ppm) and aliphatic chains (5–45 ppm) in the protein were observed to be well separated from the anomeric (91 and 104 ppm) and alcohol (55–85 ppm) carbon signals of sucrose.

When comparing the spectrum of the formulation with the one of the pure protein, no significant chemical shift differences can be observed that could imply any major changes in the structure of the protein. In contrast, the signals of the anomeric carbon atoms were clearly shifted. This can be explained by the formation of an amorphous structure of the sugar in the buffer upon freeze-drying compared to the crystalline structure of the sugar in the physical mixture. Additionally, the signal lineshapes of histidine and sucrose were found to be significantly sharper in the physical mixture compared to those observed for the formulation. This indicates phase separation of the individual components. The broad signal linewidths of both sugar and protein signals in sample F2 indicate that upon lyophilisation both components interact stronger with each other. When comparing the individual samples of lyophilised heated and unheated formulations with and without the addition of polysorbate, no significant chemical shift changes were apparent as a result of the heating or the addition of polysorbate (see ESI).

**Fig. 8:**
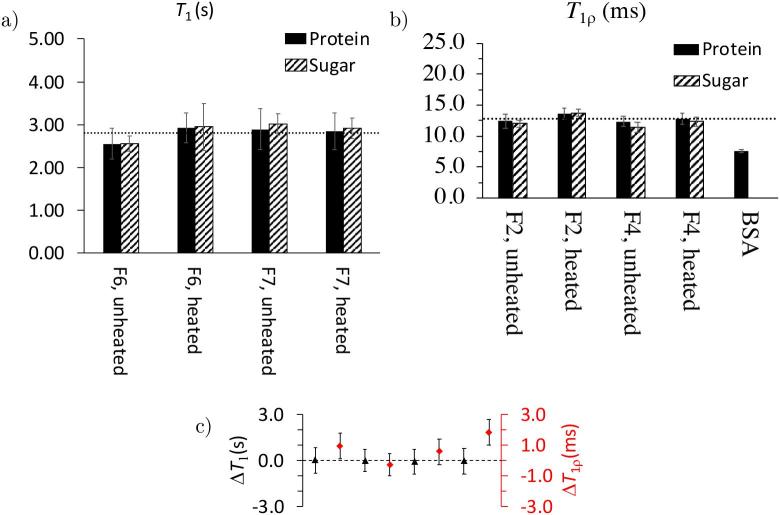
Overview of a) $T_{1}$ and b) $T_{1\rho }$ relaxation times of unheated and heated F6 and F7. The dashed line represents the average of all relaxation time values of all samples. Error bars indicate the standard error from the exponential fit. c) Difference in $T_{1}$ (left axis, black) and $T_{1\rho }$ (right axis, red) between the BSA and sucrose in samples of unheated and heated F2 and F4. The dashed line indicates ΔT=0. Error bars indicate the average error from the individual sugar and protein components. (For interpretation of the references to colour in this figure legend, the reader is referred to the web version of this article.)

Previous studies have shown that the investigation of $T_{1}$ and $T_{1\rho }$ can provide insight to changes in protein mobility in the presence of sugars (Lam et al., 2002, Yoshioka et al., 2011). By comparing the values of $T_{1}$ and $T_{1\rho }$ of the sugar and proteins, one can obtain information about phase separation in the sample on the length scale of 2–5 nm ($T_{1\rho }$) and 20–50 nm ($T_{1}$) (Mensink et al., 2016). Equal relaxation times indicate homogeneous phases, whereas different relaxation times imply separated phases. Fig. 8 shows an overview of the measured $T_{1}$ and $T_{1\rho }$ relaxation times of the formulations and pure BSA.

The values of $T_{1}$ and $T_{1\rho }$ that were observed for pure BSA are approximately a factor of 3 and 2 smaller respectively when compared to those measured for samples of F2 and F4. An increase of relaxation time in proteins is linked to the decrease of the rotational correlation time $\tau_{C}$, the time that the molecule needs to rotate by one radian (Bloembergen et al., 1948). In the current system, this indicates that the formulation contains larger average domain sizes (of lower overall mobility) due to an aggregation of the protein with the sugars. Such behaviour is in line with previous reports describing the stabilisation of proteins by sugars (Mensink et al., 2016).

The individual formulations of heated and unheated BSA and buffer with and without the addition of polysorbate show no significant differences in their relaxation times. The $T_{1}$ and $T_{1\rho }$ relaxation time differences of sucrose and BSA (Fig. 8) are the same within the error of the measurement. This indicates that no phase separation takes place within the samples upon lyophilisation, but also that heating in F2 and F4 does not cause phase separation.

The terahertz data further confirmed the information from the ssNMR measurements regarding the miscibility of sugar and protein phases as discussed above. There was no indication from the terahertz data that any phase separation had taken place (Mensink et al., 2017).

Yoshioka et al. (2011) showed that $T_{1\rho }$ measurements over a range of temperatures can be used to probe changes in molecular mobility of protein formulations. Given the considerable measurement time and the technical complexity required to measure ssNMR at low temperatures unfortunately we were only able to perform our ssNMR measurements at a static temperature.

**Fig. 9:**
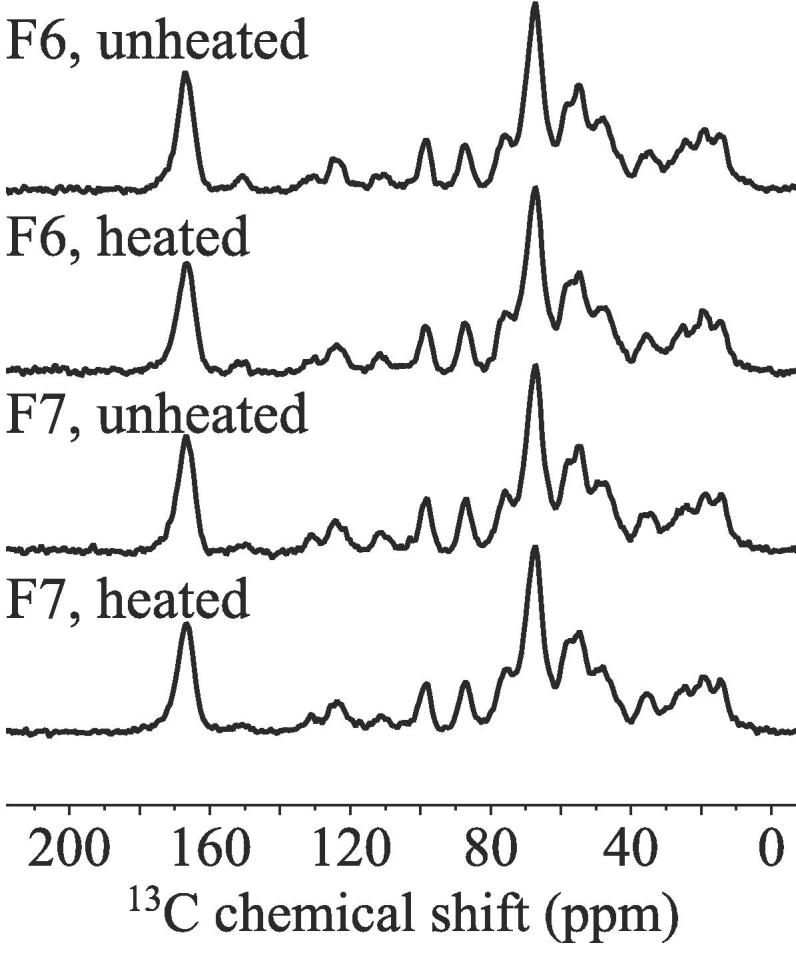
^13^C CP-MAS NMR spectra of heated and unheated F6 and F7. All the spectra were normalised to the same carbonyl signal intensity.

We acquired the ^13^C CP NMR data to detect potential structural changes in the heated and unheated samples of formulations F6 and F7 based on the acquired spectra (Fig. 9). Similar to the observations we made for NMR spectra for the samples of BSA described in the previous section no significant chemical shift changes were evident from the data when comparing the effect of heating or the addition of 0.04% w/v polysorbate.

**Fig. 10:**
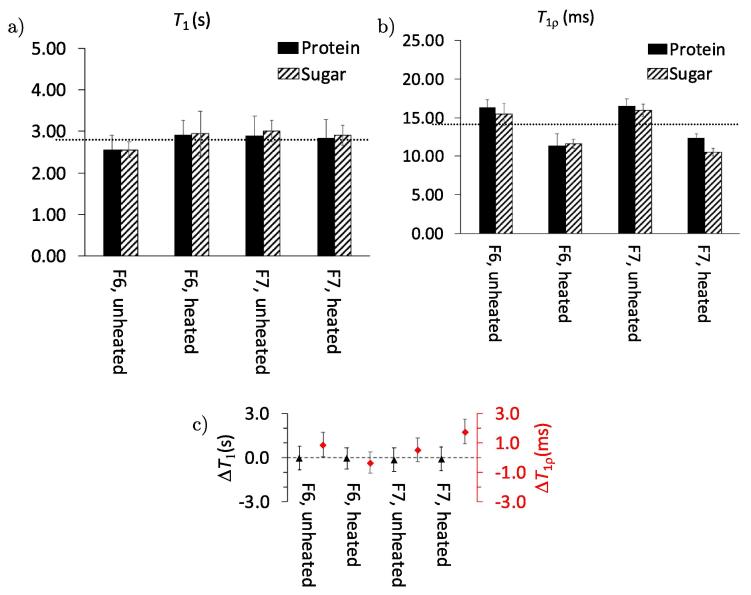
Overview of a) $T_{1}$ and b) $T_{1\rho }$ relaxation times of unheated and heated F6 and F7. The dashed line represents the average of all the respective relaxation time values of all samples. Error bars indicate the standard error from the exponential fit. c) Difference in $T_{1}$ (left axis, black) and $T_{1\rho }$ (right axis, red) between the BSA and sucrose in samples of unheated and heated F6 and F7. The dashed line indicates ΔT=0. Error bars indicate the average error from the individual sugar and protein components. (For interpretation of the references to colour in this figure legend, the reader is referred to the web version of this article.)

In addition to the ^13^C CP-MAS NMR spectra we measured the $T_{1}$ and $T_{1\rho }$ relaxation times from samples of the mAb1 formulations (Fig. 10). F6 showed a slight increase of $T_{1}$ relaxation time upon heating, whereas the value seems almost unchanged for F7 (the results were reproducible when measuring the data twice). In contrast to the results obtained for the BSA formulations, where also $T_{1\rho }$ stayed constant, the spin-lattice relaxation time decreases after heating formulations F6 and F7 (containing mAb1). As mentioned earlier, changes in $T_{1\rho }$ have previously been correlated to changes in molecular mobility by Yoshioka et al. (2011). In analogy to these results our data indicates that the molecular mobility of the protein as well as the sugars change after heating.

As discussed previously, the comparison of the relaxation times of the sugars and protein gives information about the homogeneity at different length scales (see Fig. 10c). Based on the fact that the $T_{1}$ relaxation times in the formulations for sucrose and mAb are identical it can be concluded that the samples are homogeneously mixed at a length scale of 20–50 nm. The $T_{1\rho }$ relaxation times also indicate a homogeneous phase, except for the heated F7, that indicates a partial phase separation on the 2–5 nm scales as the relaxation time of sugar and protein values are different. It is worth noting that for F6 and F7, whilst there are no differences between sugar and protein, the relaxation times for $T_{1\rho }$ are lower after heating, which is different compared to what we observe in BSA. This can be explained by considering that a decrease of $T_{1}$ relaxations times is associated with degradation. One study has showed that a significant decrease of $T_{1}$ (from 4.2 s to 3.2 s) is connected to an increased aggregation rate (from 0 to 24 a.u.) (Mensink et al., 2016). However, for the systems we investigated, we rather observe it with $T_{1\rho }$. This might be linked to a change of mobility or dynamics on a <5 nm scale. Furthermore, a variable temperature study previously probed the change of that value and linked this to different molecular mobilities (Yoshioka et al., 2011). However, this was measured at multiple temperatures and not a static one. Based on the trend observed at the static condition, the change of $T_{1\rho }$ could be connected to the change in $T_{g,\alpha }$ and this would be in correlation with the terahertz results.

## 4 Conclusions

We have examined lyophilised BSA formulations (F1-F4) and lyophilised mAb formulations (F5-F7). By performing temperature variable THz-TDS experiments we studied the structural dynamic properties of these formulations. A monotonous increase of absorption coefficient with temperature was observed for F4, F5, and F6, while the absorption coefficient plateaus at high temperatures for F1, F2, F3, and F7. All the formulations examined exhibit three temperature regimes, with a distinct $T_{g,\beta }$ and $T_{g,\alpha }$. We propose two hypotheses for the pathway protein molecules can proceed upon heating: (1) adopting different conformational states until reaching a confined state as demonstrated by a plateau in the THz-TDS data, or (2) the increase in temperature coupled with an increase in entropy leads to the protein molecules continuously exploring environments of high conformational energy and molecular mobility. Our work provides insight into the effects of excipients, including sucrose, trehalose, polysorbate 80, and arginine on the dynamics of formulations. Additionally, while the value of the transition temperatures does not change significantly between formulations, the value of the gradient does differs significantly and this is a parameter which can be obtained uniquely with THz-TDS, and could be indicative of the number of conformational environments explored by a protein system. Furthermore, we used FTIR and CD spectroscopy to investigate the structural changes of formulations and demonstrated that there is no change in the secondary structure following heating, however there is a decrease in β-sheet content of the neat BSA as excipients were incorporated in the formulations produced. Additional solid-state NMR measurements investigated changes of the formulations by ^13^C CP-MAS. A spectral analysis of the formulations showed no major chemical structural changes of the protein structures after heating. NMR relaxation time measurements of $T_{1}$ and $T_{1\rho }$ revealed a homogeneous sugar and protein phases on length scales of 2–5 nm and 20–50 nm after lyophilisation. No changes in the phase mixing were observed after heating BSA formulations containing no or 0.04% polysorbate 80. F6 and F7 show $T_{1\rho }$ decrease after heating indicating a change of dynamics in the system. The addition of 0.04% polysorbate in F7 resulted a small phase separation on the 2–5 nm scale. This work provides a framework for understanding the dynamics of complex formulations and demonstrates that THz-TDS is an effective method to measure the molecular dynamics and temperature-dependant behaviour of solid-state formulations. Furthermore, the changes in protein and excipient interactions probed by THz-TDS in the dry state may be linked to chemical stability. Specifically, systems which exhibit a plateau include proteins confined in a particular structure, which may result in exposure or protection or potential degradation sites, depending on the specific protein structure. Thus, such confined proteins may follow a different degradation pathway and/or kinetics to a non-confined protein which exhibits increasing absorption at terahertz frequencies. This relationship would provide a predictive metric for long-term chemical stability of the protein, however, additional work is required to explore these effects in detail.

## Declaration of Competing Interest

None.
