# Supplementary material for: Observation of high-temperature macromolecular confinement in lyophilised protein formulations using terahertz spectroscopy
Source: Int J Pharm X. 2019 Jul 8;1:100022. doi: 10.1016/j.ijpx.2019.100022 (PMC6733290; doi:10.1016/j.ijpx.2019.100022)
Supplement: Supplementary data [file mmc1.pdf]

Supplementary Information to Observation of High-Temperature Macromolecular Confinement in Lyophilised Protein Formulations Using Terahertz Spectroscopy

Talia A. Shmool,[†] Philippa J. Woodhams,[†] Markus Leutzsch,[†] Amberley D. Stephens ,[†] Mario U. Gaimann,[†] Michael D. Mantle,[†] Gabriele S. Kaminski Schierle,[†] Christopher F. van der Walle ,[‡] and J. Axel Zeitler[*,†]

†*Department of Chemical Engineering and Biotechnology, University of Cambridge, Philippa Fawcett Drive, Cambridge CB3 0AS, United Kingdom*

‡*Biopharmaceutical Development, AstraZeneca, Granta Park, Cambridge CB21 6GH, United Kingdom*

E-mail: jaz22@cam.ac.uk

Phone: +44 1223 334783

CD Data

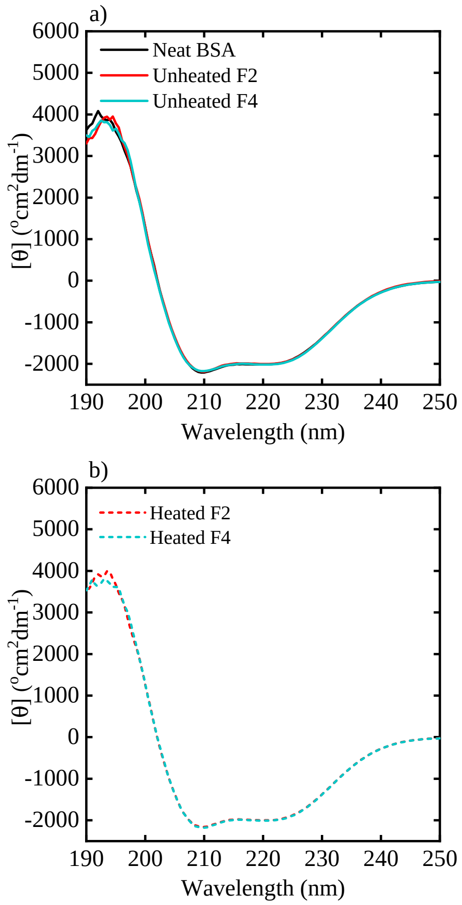

Figure S1: CD spectra of BSA. a) Black curve corresponds to the spectrum of the neat BSA, solid red curve represents F2, solid blue curve represents F3, b) dashed red curve represents heated F2, and dashed blue curve represents the heated F4.

FTIR Data

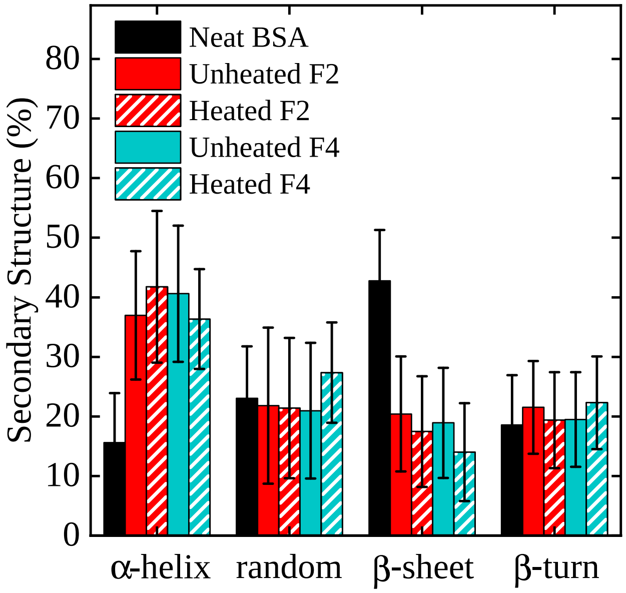

Figure S2: Secondary structure content as estimated from curve fitting to the second derivative of the FTIR spectrum. Black bar represents the neat BSA, solid red bar represents F2, solid blue bar represents F4, dashed red bar represents heated F2, and dashed blue bar represents the heated F4. Error bars represent the standard deviation for n samples: $n \geq 7$ and the intrinsic uncertainty of 5-10% from the curve fit analysis method.

Table 1: Secondary structure content as estimated from curve fitting to the second derivative of the FTIR spectrum. Errors represent the standard deviation for n samples: $n \geq 7$ and the intrinsic uncertainty of $5 - 10$ percent from the curve fit analysis method.

	α-helix (%)	Random coil (%)	β-sheet (%)	β-turn (%)
Neat BSA	15 ± 8	10 ± 9	23 ± 9	19 ± 8
Unheated F2	36 ± 10	20 ± 14	20 ± 10	22 ± 8
Heated F2	41 ± 13	21 ± 12	18 ± 9	19 ± 8
Unheated F4	40 ± 11	21 ± 11	19 ± 8	20 ± 8
Heated F4	36 ± 8	27 ± 8	14 ± 8	22 ± 8

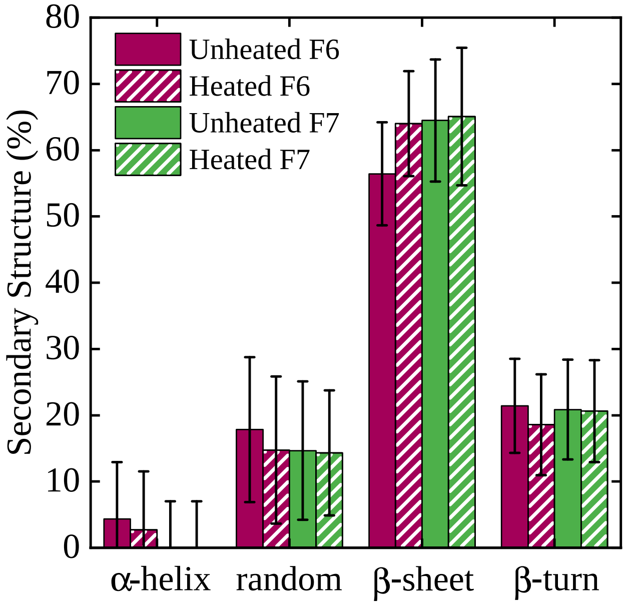

Figure S3: Secondary structure content of as estimated from curve fitting to the second derivative of the FTIR spectrum. Solid and dashed pink bars represent unheated and heated F6 respectively, solid and dashed green bars represent heated and unheated F7 respectively. Error bars represent the standard deviation for n samples: $n \geq 4$ and the intrinsic uncertainty of 5-10% from the curve fit analysis method.

Table 2: Secondary structure content as estimated from curve fitting to the second derivative of the FTIR spectrum. Error bars represent the standard deviation for n samples: $n \geq 4$ and the intrinsic uncertainty of 5-10% from the curve fit analysis method.

	α-helix (%)	Random coil (%)	β-sheet (%)	β-turn (%)
Unheated F6	4 ± 9	18 ± 11	56 ± 8	21 ± 7
Heated F6	3 ± 9	15 ± 11	64 ± 8	19 ± 8
Unheated F7	0 ± 7	15 ± 10	65 ± 9	21 ± 8
Heated F7	0 ± 7	14 ± 9	65 ± 10	21 ± 8

MDSC Data

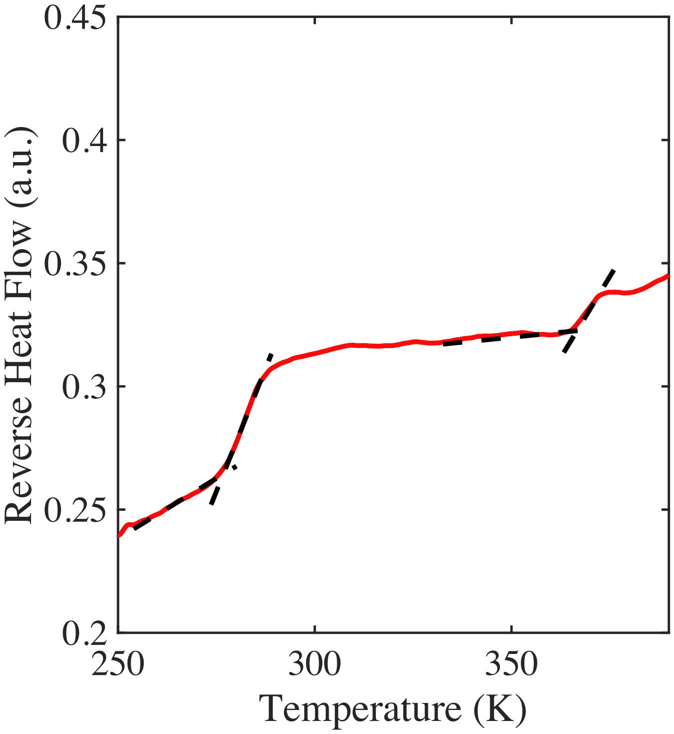

Figure S4: MDSC thermogram of sucrose and glycine mixture.

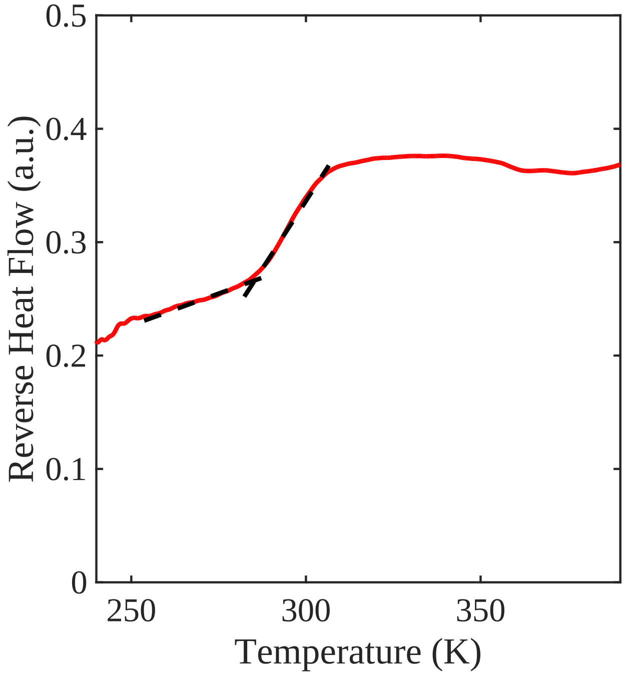

Figure S5: MDSC thermogram of F5.

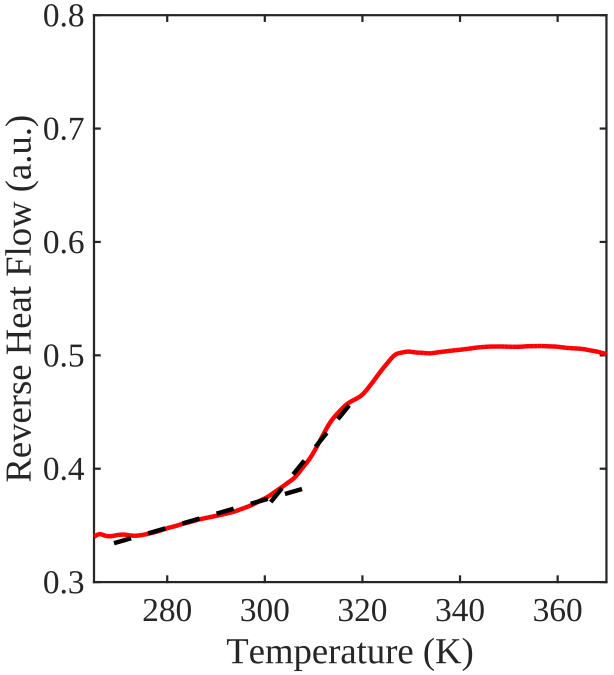

Figure S6: MDSC thermogram of F6.

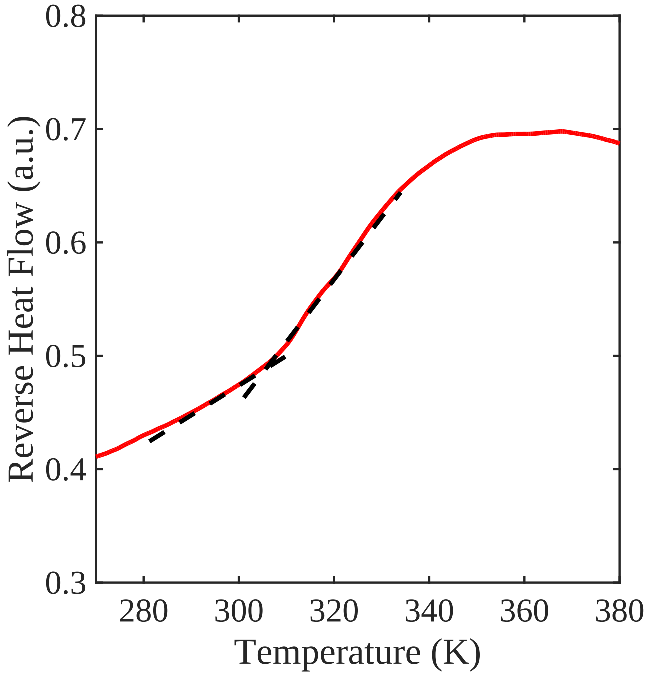

Figure S7: MDSC thermogram of F1.

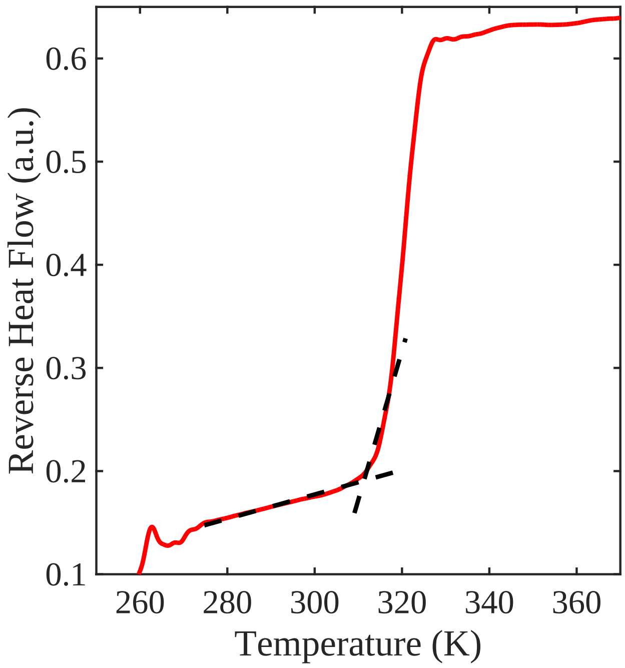

Figure S8: MDSC thermogram of F2.

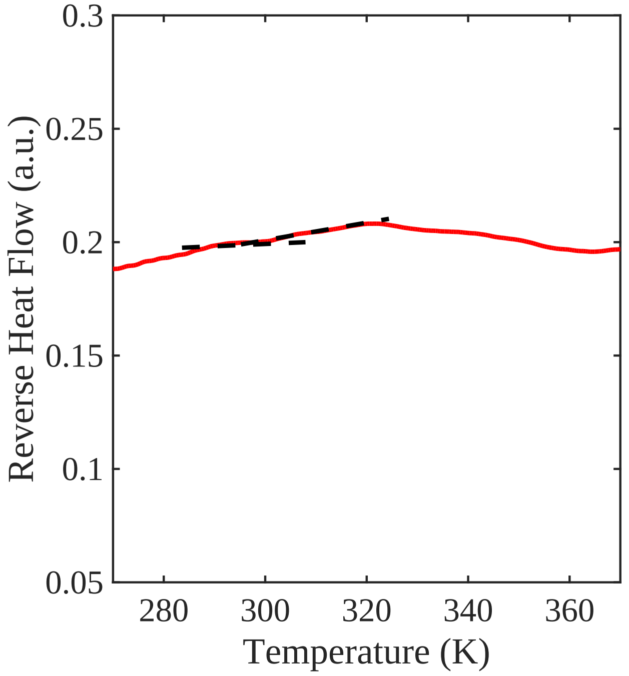

Figure S9: MDSC thermogram of F3.

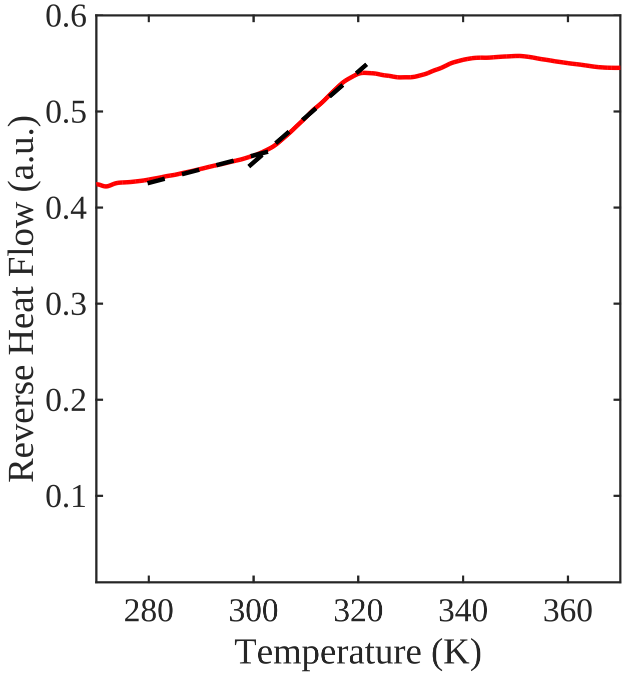

Figure S10: MDSC thermogram of F7.

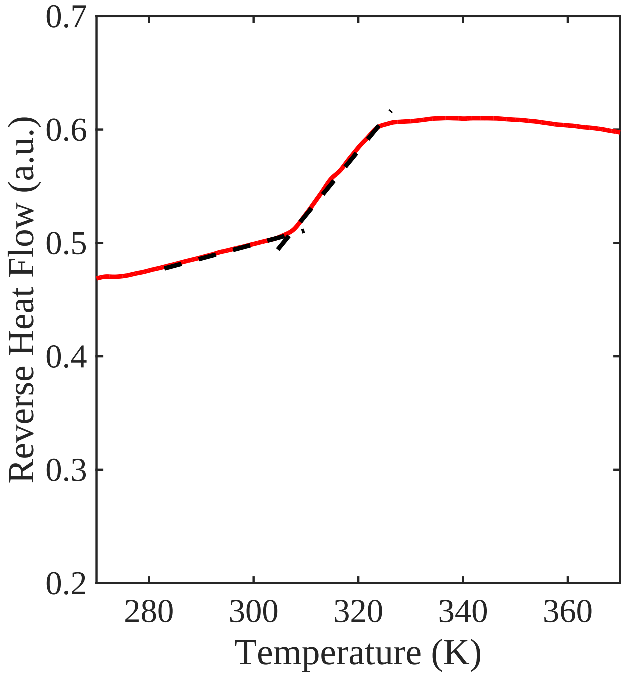

Figure S11: MDSC thermogram of F4.

Terahertz Spectroscopy Data

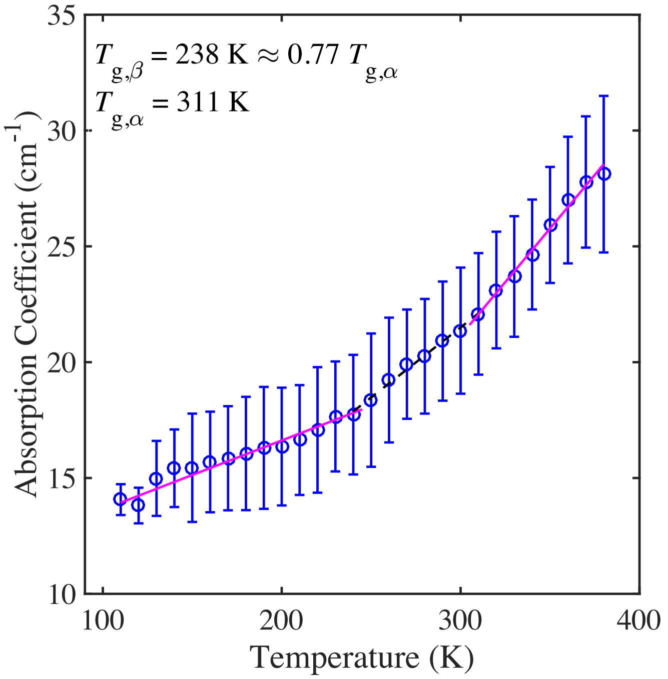

Figure S12: Terahertz absorption spectra of a physical mixture of 140 mg BSA, 8 mg Histidine, and 180 mg sucrose sample in the temperature range of $110 - 380$ K. Lines show the different linear fits for the different regions. Error bars represent the standard deviation for n samples: n = 2.

Absorption coefficient and refractive index spectra of all materials ranging between $0.2 - 2.3$ THz, in the temperature range of $100 - 440$ K, with 10 K temperature increments between spectra. Sample thickness ranged between $300 - 750$ micrometers. The absorption coefficient spectra include the upper limit of detectable absorption which is indicated by the black dashed line. Both the absorption spectra and the refractive index spectra are ordered from highest to lowest temperatures, with red lines indicating the high temperatures and blue lines indicating the low temperatures.

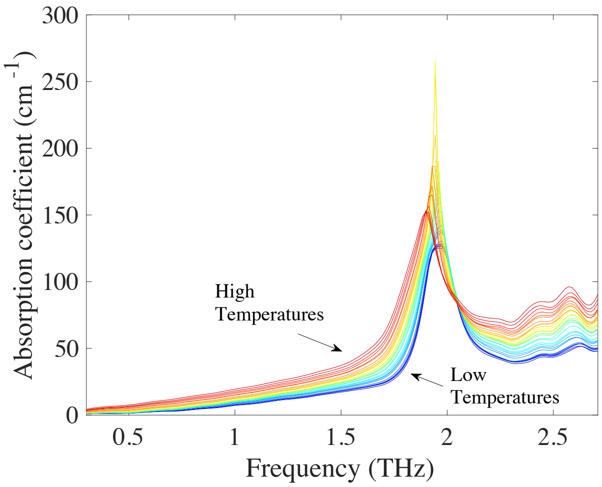

Figure S13: Absorption coefficient spectra of sucrose and glycine mixture in the temperature range of $100 - 380\,\mathrm{K}$, with $10\,\mathrm{K}$ temperature increments between spectra.

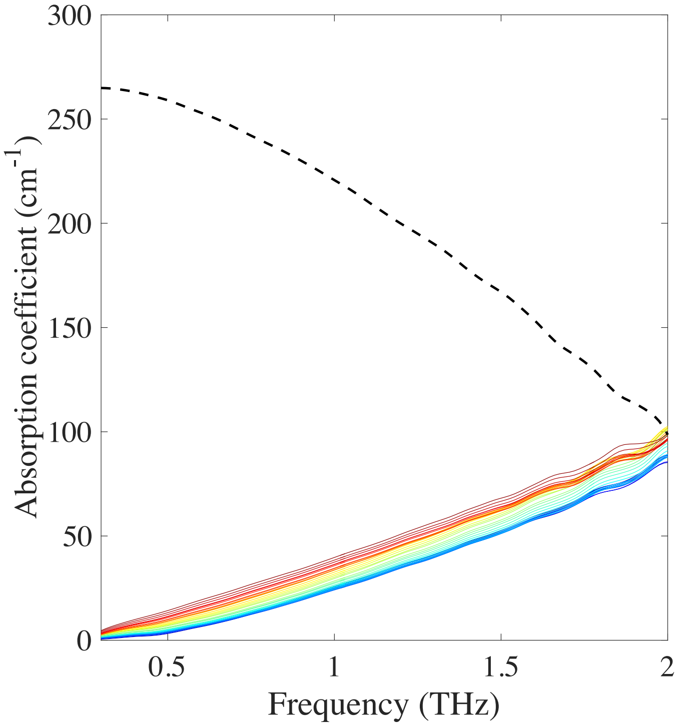

Figure S14: Terahertz absorption spectra of a F1 sample in the temperature range of $100 - 440\,\mathrm{K}$.

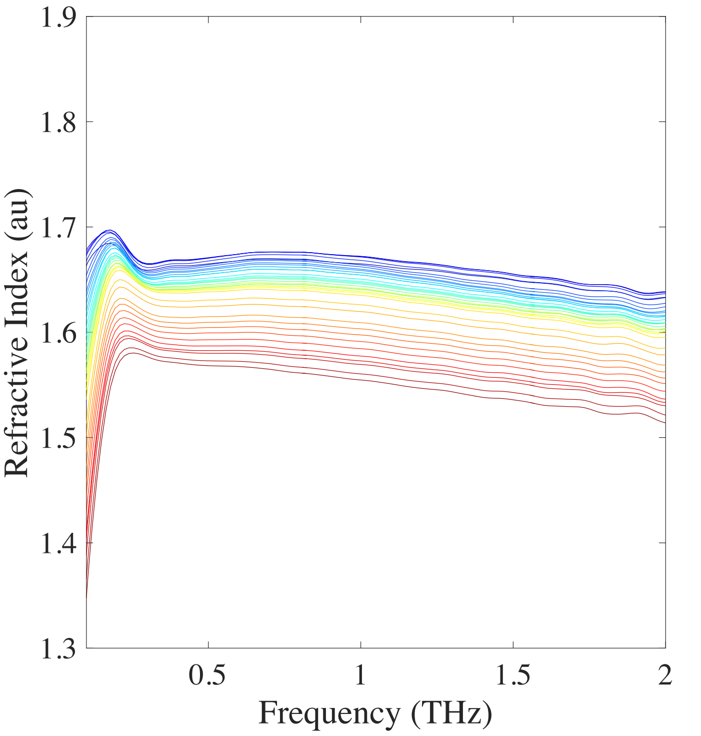

Figure S15: Refractive index spectra of a F1 sample in the temperature range of 100 − 440 K.

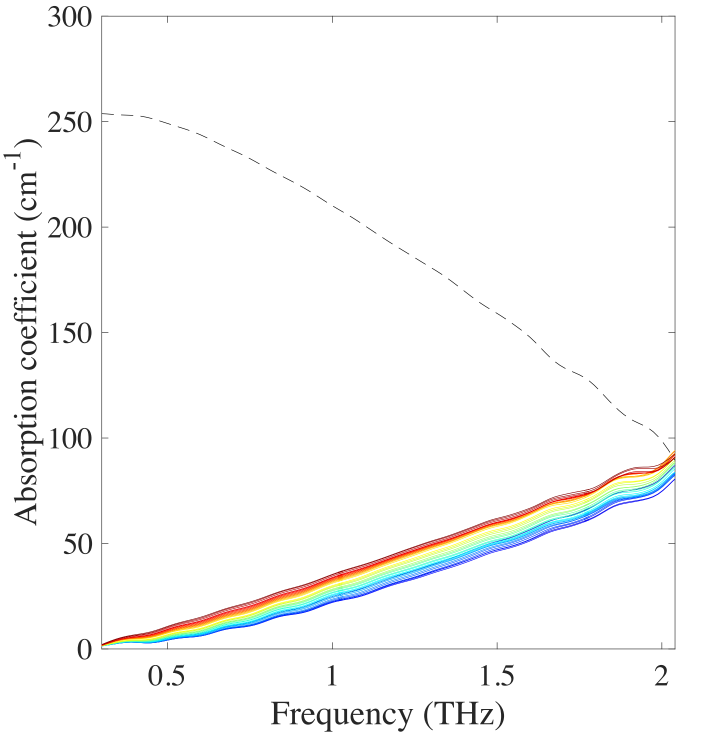

Figure S16: Terahertz absorption spectra of a F2 sample in the temperature range of 100 − 400 K.

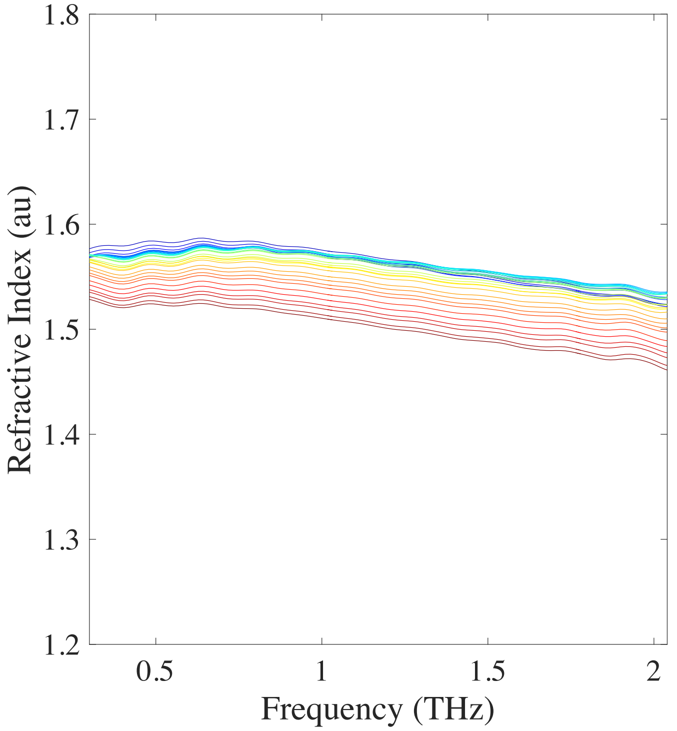

Figure S17: Refractive index spectra of a F2 sample in the temperature range of $100-400\,\mathrm{K}$.

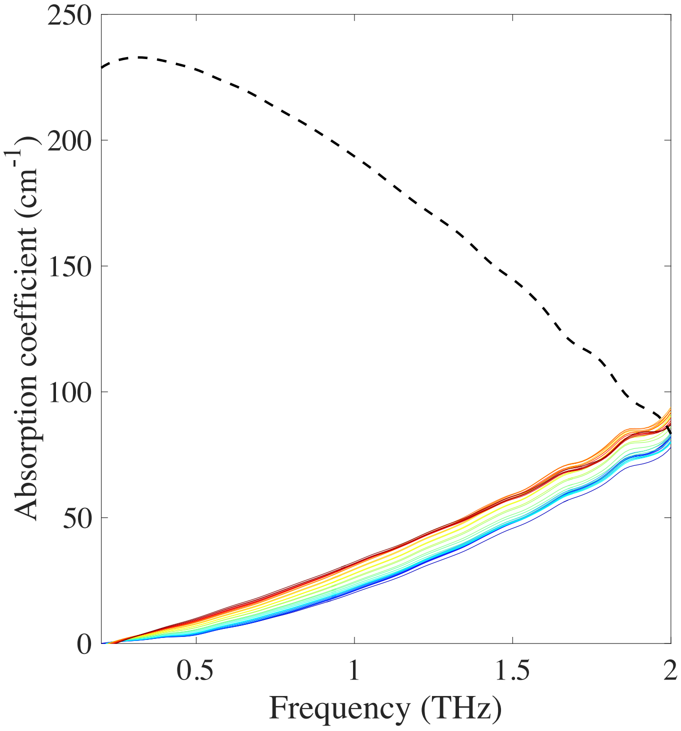

Figure S18: Terahertz absorption spectra of a F3 sample in the temperature range of $100-400\,\mathrm{K}$.

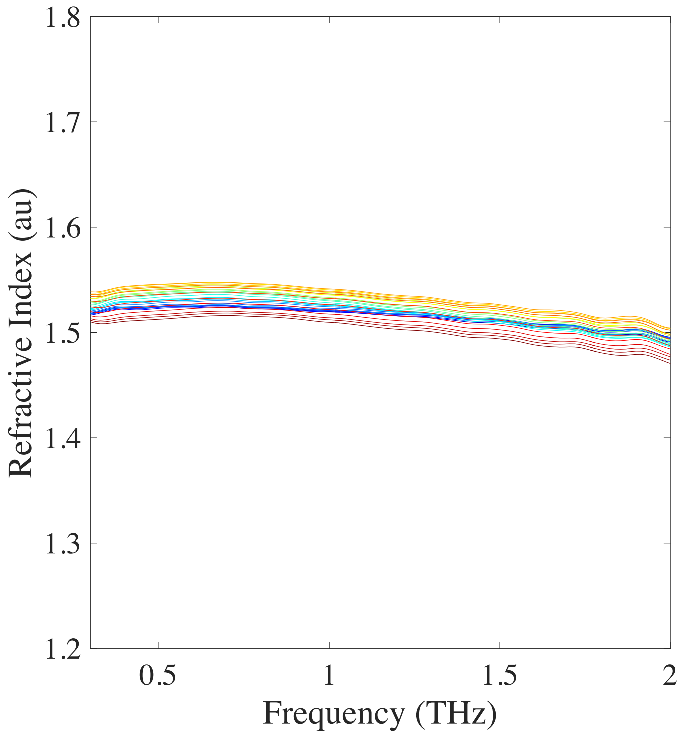

Figure S19: Refractive index spectra of a F3 sample in the temperature range of $100-400\,\text{K}$.

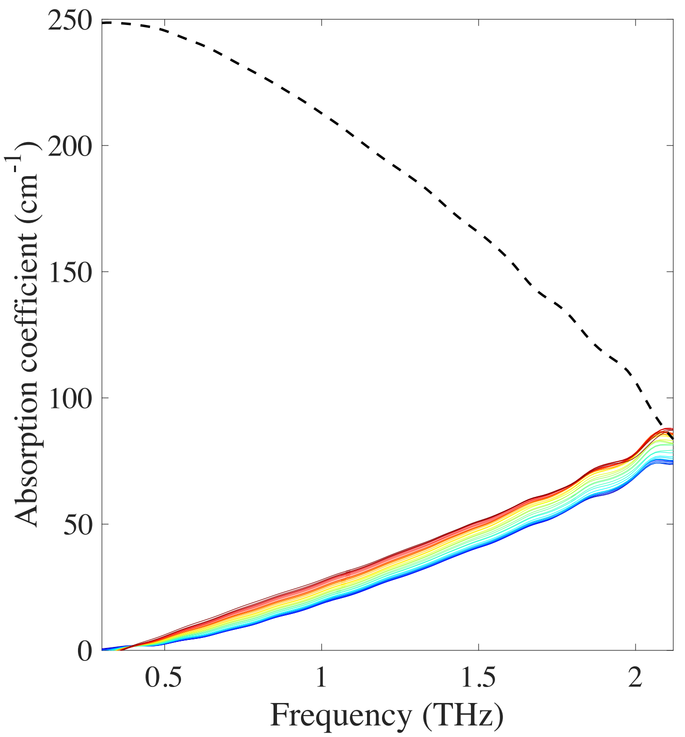

Figure S20: Terahertz absorption spectra of a F4 sample in the temperature range of $100-400\,\text{K}$.

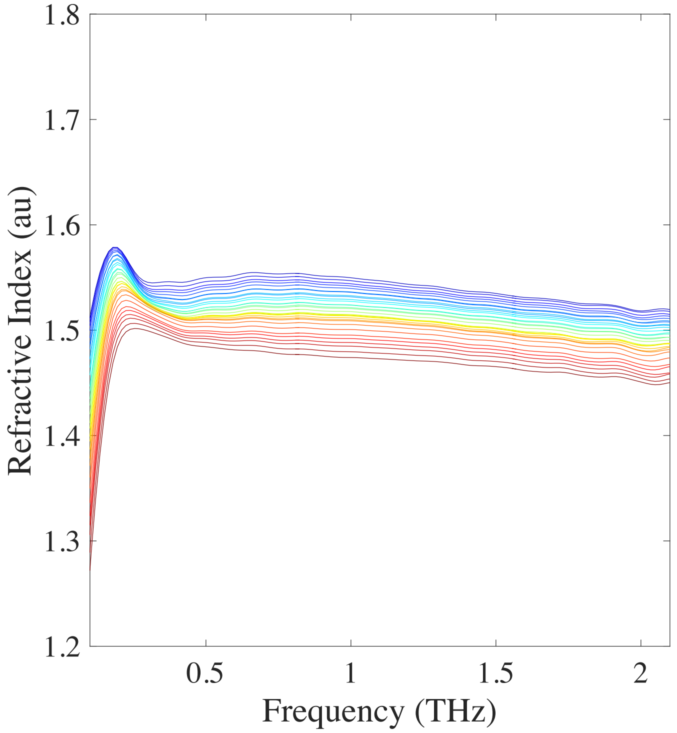

Figure S21: Refractive index spectra of a F4 sample in the temperature range of $100-400\,\mathrm{K}$.

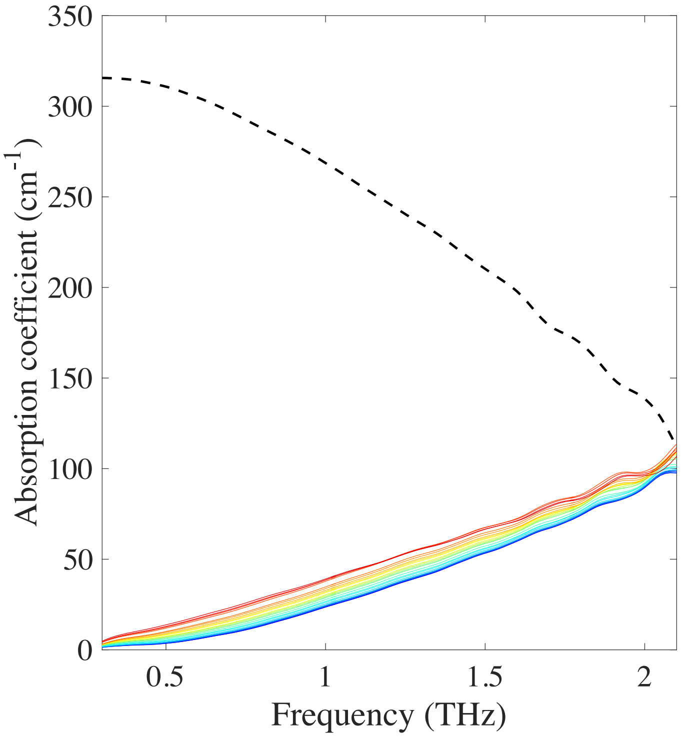

Figure S22: Terahertz absorption spectra of a F5 sample in the temperature range of $100-330\,\mathrm{K}$.

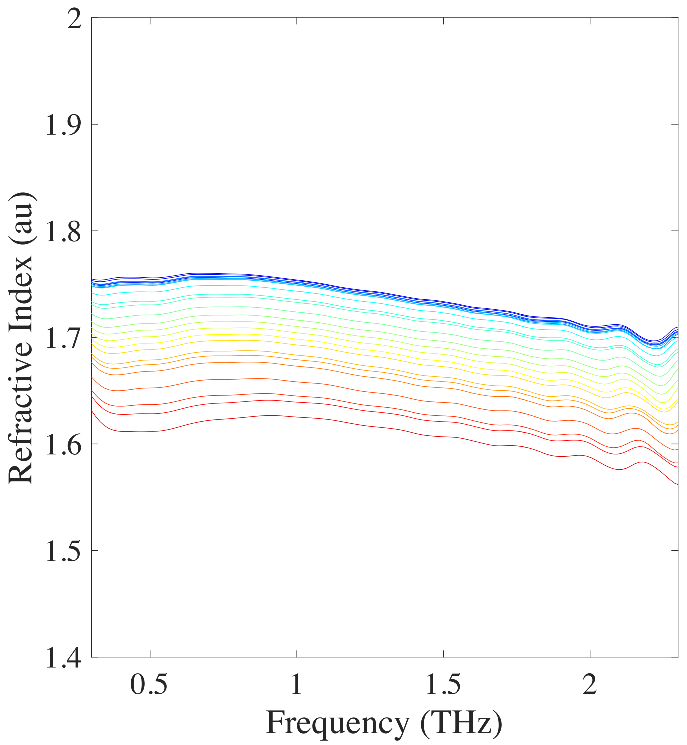

Figure S23: Refractive index spectra of a F5 sample in the temperature range of 100 − 330 K.

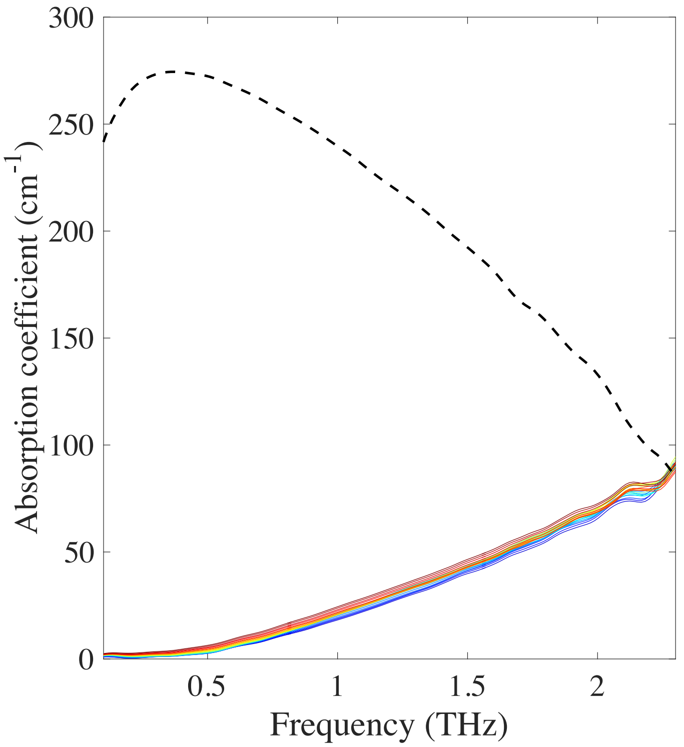

Figure S24: Terahertz absorption spectra of a F6 sample in the temperature range of 110 − 330 K.

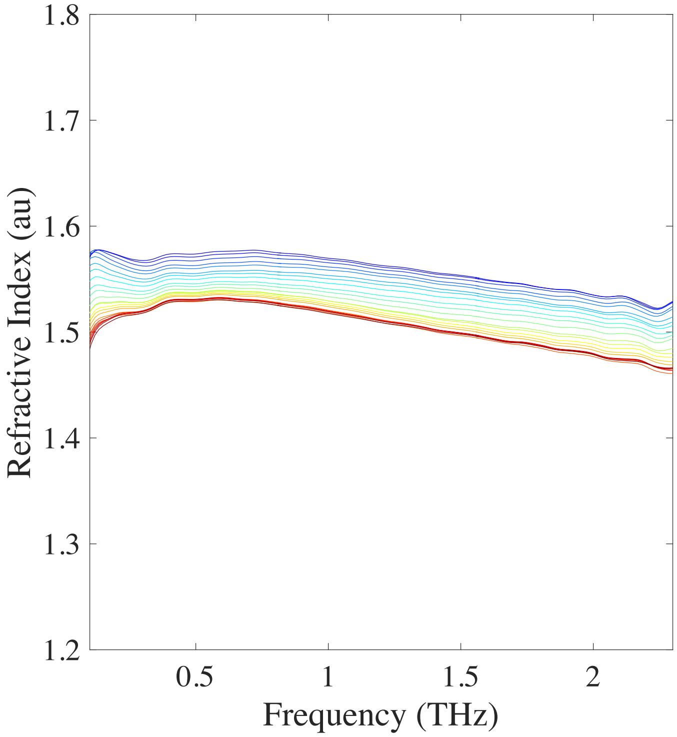

Figure S25: Refractive index spectra of a F6 sample in the temperature range of 110 − 330 K.

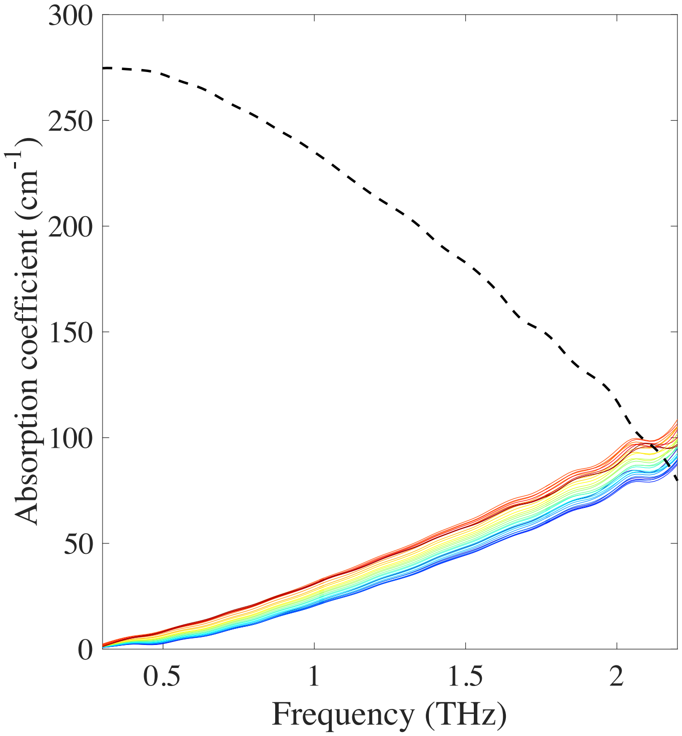

Figure S26: Terahertz absorption spectra of a F7 sample in the temperature range of 110 − 370 K.

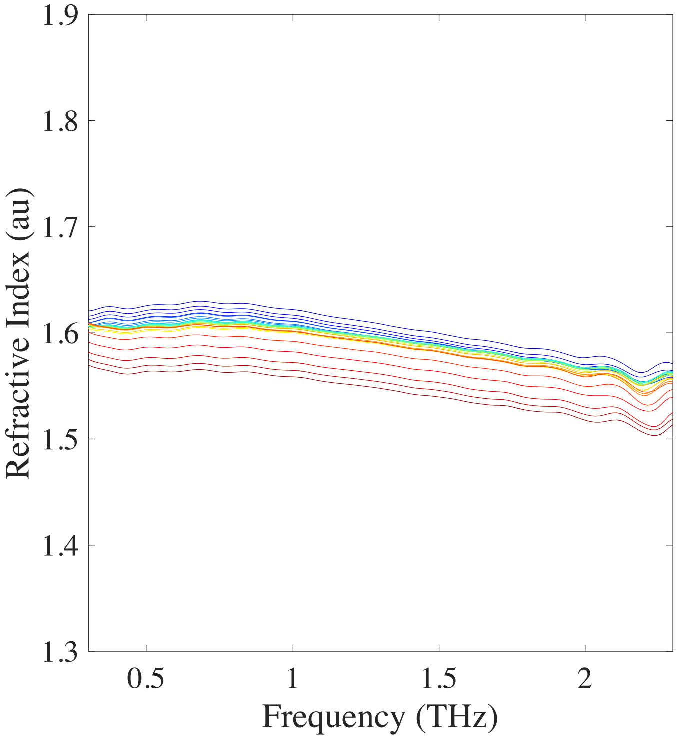

Figure S27: Refractive index spectra of a F7 sample in the temperature range of $110-370\,\mathrm{K}$.

NMR Data

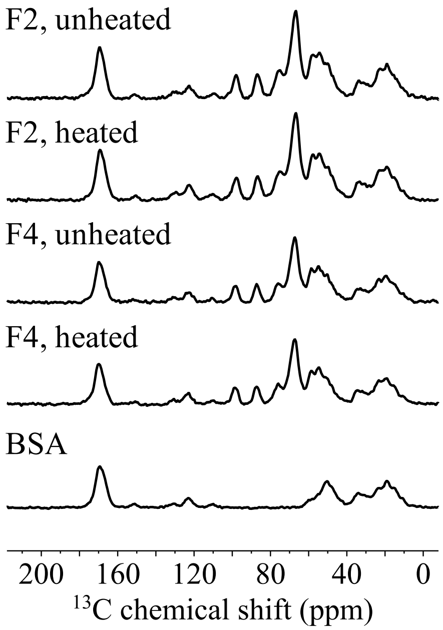

Figure S28: ^{13}C CP-MAS NMR spectra of heated and unheated F2 and F4 compared to BSA as received from the supplier. All spectra were normalised to the same carbonyl signal intensity.
